# Recent advances in biopolymer-based hemostatic materials

**DOI:** 10.1093/rb/rbac063

**Published:** 2022-09-21

**Authors:** Marvin Mecwan, Jinghang Li, Natashya Falcone, Menekse Ermis, Emily Torres, Ramon Morales, Alireza Hassani, Reihaneh Haghniaz, Kalpana Mandal, Saurabh Sharma, Surjendu Maity, Fatemeh Zehtabi, Behnam Zamanian, Rondinelli Herculano, Mohsen Akbari, Johnson V. John, Ali Khademhosseini

**Affiliations:** Terasaki Institute for Biomedical Innovation, Los Angeles, CA 90064, USA; Terasaki Institute for Biomedical Innovation, Los Angeles, CA 90064, USA; Terasaki Institute for Biomedical Innovation, Los Angeles, CA 90064, USA; Terasaki Institute for Biomedical Innovation, Los Angeles, CA 90064, USA; Terasaki Institute for Biomedical Innovation, Los Angeles, CA 90064, USA; Department of Molecular and Cell Biology, University of California, Berkeley, CA 94720, USA; Terasaki Institute for Biomedical Innovation, Los Angeles, CA 90064, USA; Department of Biomedical Engineering, University of Southern California, Los Angeles, CA 90089, USA; Terasaki Institute for Biomedical Innovation, Los Angeles, CA 90064, USA; Terasaki Institute for Biomedical Innovation, Los Angeles, CA 90064, USA; Terasaki Institute for Biomedical Innovation, Los Angeles, CA 90064, USA; Terasaki Institute for Biomedical Innovation, Los Angeles, CA 90064, USA; Terasaki Institute for Biomedical Innovation, Los Angeles, CA 90064, USA; Terasaki Institute for Biomedical Innovation, Los Angeles, CA 90064, USA; Terasaki Institute for Biomedical Innovation, Los Angeles, CA 90064, USA; Terasaki Institute for Biomedical Innovation, Los Angeles, CA 90064, USA; Bioengineering & Biomaterials Group, School of Pharmaceutical Sciences, São Paulo State University (UNESP), Araraquara, SP 14800-903, Brazil; Terasaki Institute for Biomedical Innovation, Los Angeles, CA 90064, USA; Department of Mechanical Engineering, University of Victoria, Victoria, BC V8P 5C2, Canada; Biotechnology Center, Silesian University of Technology, Gliwice 44-100, Poland; Terasaki Institute for Biomedical Innovation, Los Angeles, CA 90064, USA; Terasaki Institute for Biomedical Innovation, Los Angeles, CA 90064, USA

**Keywords:** hemostasis, coagulation, polysaccharides, polypeptides, biomaterials

## Abstract

Hemorrhage is the leading cause of trauma-related deaths, in hospital and prehospital settings. Hemostasis is a complex mechanism that involves a cascade of clotting factors and proteins that result in the formation of a strong clot. In certain surgical and emergency situations, hemostatic agents are needed to achieve faster blood coagulation to prevent the patient from experiencing a severe hemorrhagic shock. Therefore, it is critical to consider appropriate materials and designs for hemostatic agents. Many materials have been fabricated as hemostatic agents, including synthetic and naturally derived polymers. Compared to synthetic polymers, natural polymers or biopolymers, which include polysaccharides and polypeptides, have greater biocompatibility, biodegradability and processibility. Thus, in this review, we focus on biopolymer-based hemostatic agents of different forms, such as powder, particles, sponges and hydrogels. Finally, we discuss biopolymer-based hemostatic materials currently in clinical trials and offer insight into next-generation hemostats for clinical translation.

## Graphical Abstract

**Figure:**
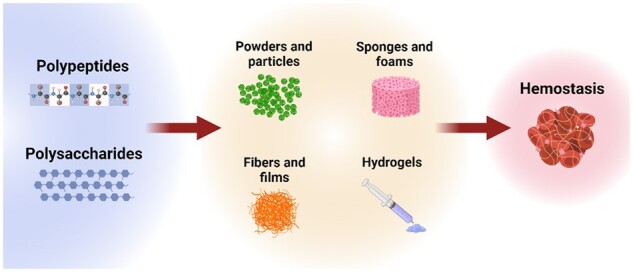

## Introduction

Hemorrhage is the loss of blood from blood vessels due to traumatic injuries and is responsible for 30–40% of trauma-related mortality [1–3]. Hemostasis is the first step in the wound healing process and is the body’s natural mechanism to stop bleeding. In response to an injury, blood vessels vasoconstrict to restrict blood flow, and the damaged endothelial cells release von Willebrand factor to trigger a complex clotting factor cascade to form a soft platelet plug, followed by a stronger fibrin clot [4]. Often, the process of hemostasis starts within seconds of an injury, and it can take up to several minutes for the fibrin clot to be formed [5]. Depending on the type of injury, the individual may lose excessive amounts of blood and experience hemorrhagic shock due to inadequate oxygen and nutrient delivery to tissue [2]. To avoid excessive blood loss, hemostatic agents are needed to facilitate coagulation.

An ideal hemostat for emergency settings, battlefield injuries and premedical treatment facility hemorrhage control should have the following characteristics [6–9]: (i) quick and adequate hemostasis in a wide range of injuries and wounds; (ii) sustained hemostasis for several hours with the ability to deliver antibiotics in situations of delayed evacuation; (iii) easy removal without any residues left in the injury or wound; (iv) ready-to-use and ease-of-administration by a layperson with little to no training; (v) ease-of-manufacturing and sterilization with low cost; (vi) easy storage with prolonged stability even under austere conditions; (vii) good biocompatibility with low/no adverse body immune-response. In emergency medicine, several types of hemostatic agents have been employed, including chemical or topical hemostats [10, 11], direct pressure or pressure dressings [12], sutures and ties [13, 14], as well as physical agents [15], and they sometimes need to be combined to achieve effective hemostasis [16, 17].

The field of hemorrhage control is expansive and quickly evolving. This review summarizes the recent advances in biopolymer-based materials for hemostatic applications. At first, we describe the coagulation cascade, the various pathways and clotting factors involved in the clot formation. Then, we provide insights into different strategies to achieve coagulation and stop bleeding, focusing on using fibrin, thrombin, clotting factors and molecular drugs. Notably, we explore a great range of polysaccharide- and polypeptide/protein-based materials and their composites for bleeding management. Specifically, biopolymer-based hemostats of several forms, including powder and particles, sponges and foams, sheets and films, and hydrogels, and their importance for specific hemostatic applications have been reviewed. Finally, we discuss biopolymer-based hemostats currently in clinical trials and provide an outlook on where the field of hemostasis is headed.

## Hemostasis coagulation cascade

Hemostasis is the natural mechanism in response to vascular endothelial impairment. It involves a series of events supporting the production of a clot to preserve blood within the vasculature [18]. There are two types of hemostasis: primary and secondary. Vasoconstriction and platelet adhesion, activation and aggregation are all involved in primary hemostasis, which leads to the creation of a soft platelet plug. This process is then followed by secondary hemostasis, which results in the transformation of a soft platelet plug into a hard, insoluble fibrin clot achieved by the conversion of fibrinogen to fibrin [19].

**Figure 1.**
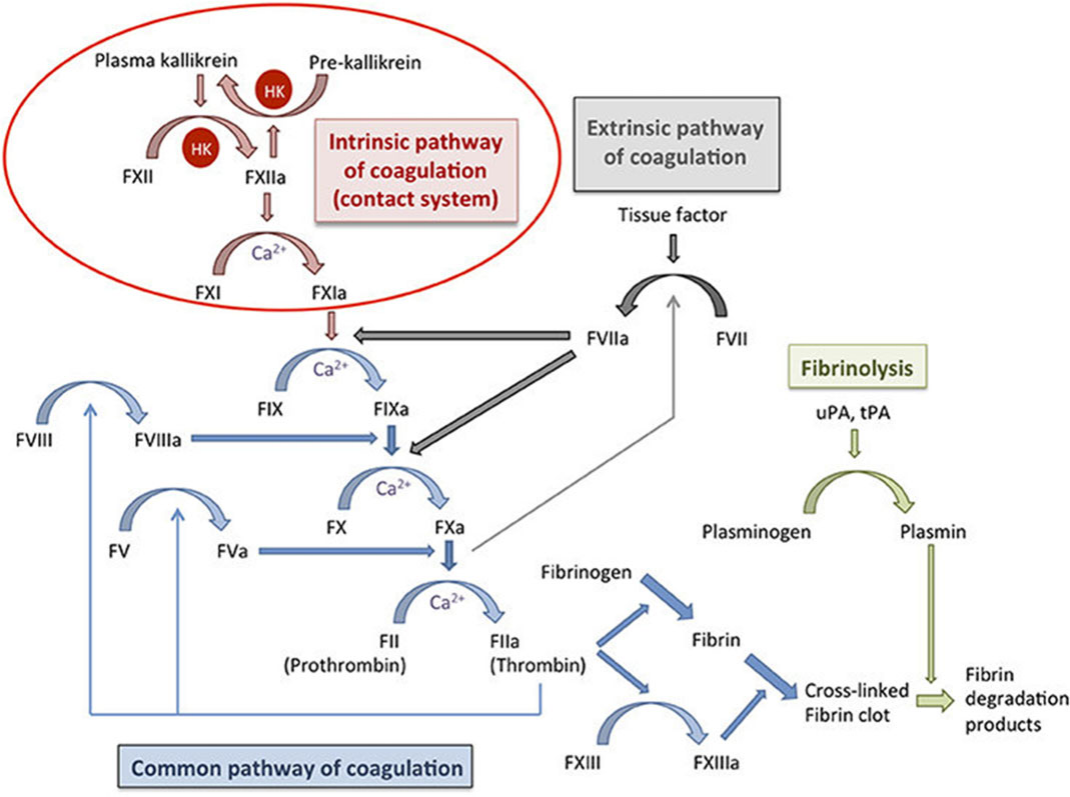
The schematic representation of the coagulation cascade, which includes the intrinsic pathway, extrinsic pathway and common pathway. Both the intrinsic and extrinsic pathways converge at the common pathway and lead to factor X activation, which helps convert prothrombin to thrombin. Thrombin is necessary to convert fibrinogen to fibrin and activate factor XIII, which eventually leads to a crosslinked fibrin clot. Fibrinolysis helps regulate hemostasis and helps degrade the fibrin network via plasmin. HK, high molecular weight kininogen; uPA, urokinase plasminogen activator; tPA, tissue plasminogen activator. Image adapted with permission from Loof *et al.* [29].

The mechanism of hemostasis coagulation is a complicated process involving many clotting factors, such as fibrinogen, prothrombin, etc., that activate each other and create a cascade (Fig. 1). Blood clotting factors are critical elements of hemostasis. They circulate in the blood in an inactivated form known as zymogens or proenzymes [20]. The coagulation process highly depends upon the amount of these proteins in the blood [21]. Majority of the coagulants are produced in the liver except for a few [22]. Most clotting factors are produced primarily through hepatocytes, which play a major role in delivering a variety of clotting factors (XIII, XII, XI, X, IX, VII, V, II and I). On the other hand, clotting factors VIII (antihemophilic factor A) and III (tissue factor) are produced by endothelial cells, whereas clotting factor IV (calcium ion) is found freely in plasma. Megakaryocytes are responsible for the development of platelets as well as component V [23, 24].

Traditionally, the coagulation cascade is categorized into two major pathways based on activation mechanism: intrinsic and extrinsic. The intrinsic pathway is engaged in response to internal vascular endothelial injury, whereas the extrinsic pathway is triggered in response to extrinsic trauma. These two pathways converge into a common pathway at the fibrin clotting step with factor X (FX) activation. Activated clotting factors are denoted by adding ‘a’ at the end of the name.

### Intrinsic pathway

The intrinsic pathway is considered as the initiation of the coagulation reaction process, involving factors I (fibrinogen), II (prothrombin), IX (Christmas factor), X (Stuart-Power factor), XI (plasma thromboplastin) and XII (Hageman factor) (Fig. 1). The intrinsic pathway is initiated by the autoactivation of FXII (factor XII), which changes its conformation. Negatively charged molecules or surfaces can activate FXII [22]. After hemorrhage, FXII is activated by either negatively charged collagen fibrils in damaged tissue or negatively charged phospholipids from endothelial cells. Activated FXII (FXIIa) converts factor XI to FXIa. Next, downstream, FXIa further activates factor IX to FIXa. FIXa subsequently activates factor X by combining with its cofactor VIIIa, which together forms a complex on a phospholipid surface. FXa binds to FVa and converts prothrombin to thrombin. In addition, FXIIa converts prekallikrein to kallikrein, which in turn activates FXII and maintains a feedback loop to amplify the production of FXIIa.

### Extrinsic pathway

The extrinsic pathway is shorter compared to the intrinsic pathway. This pathway involves factors I, II (Prothrombin), VII (Stable factor) and X (Fig. 1). It is activated by vascular injury when endothelial cell-released tissue factors (TFs) located in the extracellular matrix bind to factor VII to form FVIIa and initiate the extrinsic pathway cascade [25]. FVIIa and TF bind to FX (FXa), which binds to factor V and activates (Va). FXa binds to FVa, and calcium (Ca^2+^) produces a prothrombinase complex entering the common pathway [22].

### Common pathway

The common pathway involves factors I, II, V, VII and X (Fig. 1). It initiates after activating factor X (FXa) via the intrinsic pathway form a complex with factors VIII, IXa, a phospholipid and Ca^2+^ or via the extrinsic pathway involving factors VII, TF and Ca^2+^ [26]. After activating factor Xa, it converts factor II to factor IIa with cofactor V. This complex formation occurs at the cell membrane of the platelet with phosphatidylserine. Factor IIa activates other factors and cofactors such as FXI, V, VIII and XIII, creating a feedback loop. Factor IIa further converts fibrinogen to fibrin leading to fibrin polymerization. This reaction is catalyzed by factor XIIIa. Polymerized fibrin further creates a network structure by crosslinking with FXIIIa, activating lysine and glutamic acid. This reaction further stabilizes the platelet plug leading to activation and aggregation, creating a thrombus. This fibrin network structure is the primary structure of the blood clot [18].

The coagulation cascade is controlled by other factors that encounter the coagulation activity. For example, antithrombin (AT III) inactivates IIa and FXa by blocking the enzyme (i.e. serine protease) activity at the injured site, forming a complex structure. The process is slower and can be counteracted by using heparin [27]. Moreover, in specific clinical cases, limitations of the coagulation cascade are observed. Patients deficient in coagulation factor FXII, which is the initiation factor of intrinsic pathways, can have thromboplastin partially without bleeding tendency. In contrast, a patient deficient in factors VII, VIII and IX, shows bleeding tendency while extrinsic pathways remain unaffected [28]. This shows that the intrinsic and extrinsic pathways are dependent [21].

## Targeting strategies for achieving hemostasis

As pointed out in the previous section, hemostasis is a tightly regulated process that results in coagulation, owing to the synchronized action of enzymes and clotting factors. Here, it is worth mentioning that in instances of severe blood loss, adopting adjuvant measures for bleeding control becomes necessary since the body's natural mechanism cannot control hemostasis on its own. Therefore, understanding the natural hemostasis process is essential to developing practical hemostatic devices that can mimic and stimulate the natural process [30].

Fibrin is one of the most popular natural hemostatic agents. It is formed from fibrinogen under the action of thrombin at a wound site or a site of tissue damage in the presence of factor XIII and calcium and, in combination with platelets, creates a blood clot [31]. However, during the shortage of clotting factors, either due to high blood loss, genetic diseases or excessive use of anticoagulants, thrombin activation is inhibited, which leads to inhibition of fibrin formation [6]. The thickness of the layer of fibrin fibers solely depends on the thrombin concentration. Moreover, the weak and thin fibrin fibers are more prone to fibrinolysis, hindering hemostasis [32]. Therefore, there are several strategies to use biomaterials to create fibrin fibers by mimicking clotting factors and inducing mechanically strong clot formation for hemostasis applications [33].

The synthetic peptide mimicking knob A, such as double-headed ligand bis(Gly-Pro-Arg-Pro-amido)polyethylene-glycol, was first reported to be the replacement for thrombin-modified-E-nodule for fibrinogen–fibrinogen or D–D crosslinking (non-covalent) by the interaction between the two-hole a’s in the γ-chain in the vicinity [34]. As a result, the peptide was found to crosslink the fibrin by showing the biphasic behaviors (productive and non-productive) depending on the peptide concentration [35]. Later, cysteine-terminated knob-A-peptide mimics (GPRPAAC) were conjugated with four-arm and two-arm maleimide-functionalized polyethylene glycols (PEGs) to synthesize GPRP_4_-PEG. At a lower concentration of GPRP_4_-PEG to fibrinogen, the clotting showed higher density and larger fiber diameter [36]. On the contrary, PEGylated knob peptides were recognized as anticoagulants [37]. In addition, the linear polymer hemostats (PolySTAT), composed of poly(hydroxyethyl)methacrylate conjugated to *N*-hydroxysuccinimidyl ester methacrylate, were also found to crosslink fibrin. The PolySTAT-crosslinked fibrin was denser and less porous with higher elastic moduli than the control [38–40]. For the rat model, the intravenous injection of PolySTAT showed a significantly higher survival rate for the injury at the femoral artery [38].

Apart from that, engineered platelet-like-particles (PLPs) composed of ultra-low-crosslinked poly(N-isopropyl acrylamide-co-acrylic acid) microgel particles with single domain variable fragments (sdFv’s) were developed for use in intravenous hemorrhage in the case of injury in a rat model. The PLPs deformed like platelets during fibrin formation due to the low density of crosslinking [41]. Besides, the nanoparticles created by cholic-acid-mediated self-assembly of polyethyleneimine release various soluble biomolecules, including growth factors, coagulant factors and extracellular vesicles. Platelets can also evade the immune system, adhere to the subendothelial layer and interact with pathogens [42, 43]. Due to these properties, numerous platelet membrane-coated nanostructures have been developed as drug delivery methods. Numerous scientific groups around the world demonstrated that drug-containing silica or copolymer nanoparticles could be targeted with membranes using platelets extracted from the whole blood of humans. These platelet-like particles could be fabricated in the form of biomimetic nanocarriers [44, 45].

Drugs, polymers and technological advances that inhibit fibrinolysis or improve fibrin strength (and stability) can be of considerable therapeutic benefit. Tranexamic acid (TXA) is an US Food and Drug Administration (FDA) approved synthetic derivative of the amino acid lysine. TXA is responsible for downregulation of upregulated tissue plasminogen activator, fibrinolysis by inhibiting the lysine-binding sites on plasminogen, treats severe menstrual and postpartum bleeding, and trauma therapy. Several clinical research findings suggest that TXA is also linked to off-target systemic coagulopathy, thromboembolic consequences and neuropathy [46–50]. Nanomedicine-based platforms can resolve these obstacles by either (i) encapsulating TXA within the liposomal nanovesicles, (ii) actively targeted surface functionalization with a cyclo-CNPRGDY(OEt)RC peptide and (iii) using fibrinogen-mimetic peptide targeted on integrin GPIIb-IIIa for anchoring to active platelets within trauma-associated clots. Off-target effects can be avoided by utilizing targeted delivery of TXA to the traumatic injury site via liposomes, enabling its clot-stabilizing action to improve hemostasis and survival [32, 46].

Researchers recently recommended that super-hydrophobic or super-hydrophilic materials be used for hemostatic reasons. It has been observed that a super-hydrophobic graphene sponge quickly absorbs water from the blood, generating a thick layer of blood cells and platelets and thus increasing coagulation [51]. Spray-coated chitosan on the nanopores hollow-fibrous matting may also produce a hydrophilic hemostatic product, and the hydrophilic chitosan coating can improve blood surface properties and coagulation. Moreover, the fundamental operation of these techniques has been predicated on blood-absorbing (super-hydrophilic) hemostatic substances that reduce internal and peripheral bleeding or blood-repelling (super-hydrophobic) materials that simply resist blood but do not effectively trigger clot formation. Super-hydrophobic carbon nanotubes fibrous gauze were developed to overcome these problems, achieving fast clotting without blood loss [51]. These hemostatic material surfaces tend to detach from the clot following clot maturation, driven by the contractile tension in the clot contraction phase, allowing a natural and unforced removal of the hemostatic dressing without inducing subsequent hemorrhage. Topical patches or gauzes fabricated by micro/nanoparticles or fibers within bounded polymer networks prevent loose micro/nanoparticles or fibers from entering the vascular system [51]. These technologies have proven to be safe and compatible, can minimize microbial contamination and lower the chance of infection.

Hemophilia A is a bleeding disorder caused by hereditary coagulation factor VIII deficiency (FVIII). The conventional injectable clotting factor augmentation was formerly used two or three times per week to treat hemophilia, with low patient compliance, high costs and the formation of inhibitory antibodies following long-term treatment. Novel gene-based treatment has previously been explored and found to be a promising treatment for hemophilia. For example, recombinant FVIII (N8-GP) has an extended half-life, lesser reactivity and better hemostasis effectiveness. Some of those are appealing, new techniques with promising clinical uses, but they have certain efficacy and safety limitations. Because of individual variances in FVIII activity, the typical dose regimen of recombinant FVIII must be further formed to ensure the regulation of supplemental proteins in plasma for efficiently minimizing bleeding. However, in a recent study, asialoglycoprotein receptor ligand (*N*-acetylgalactosamine [GalNAc]-heparin cofactor II [HCII]) was conjugated to small interfering RNA, allowing for active targeting to the liver targeting the heparin cofactor II [52].

## Polysaccharide materials for hemostasis

**Figure 2.**
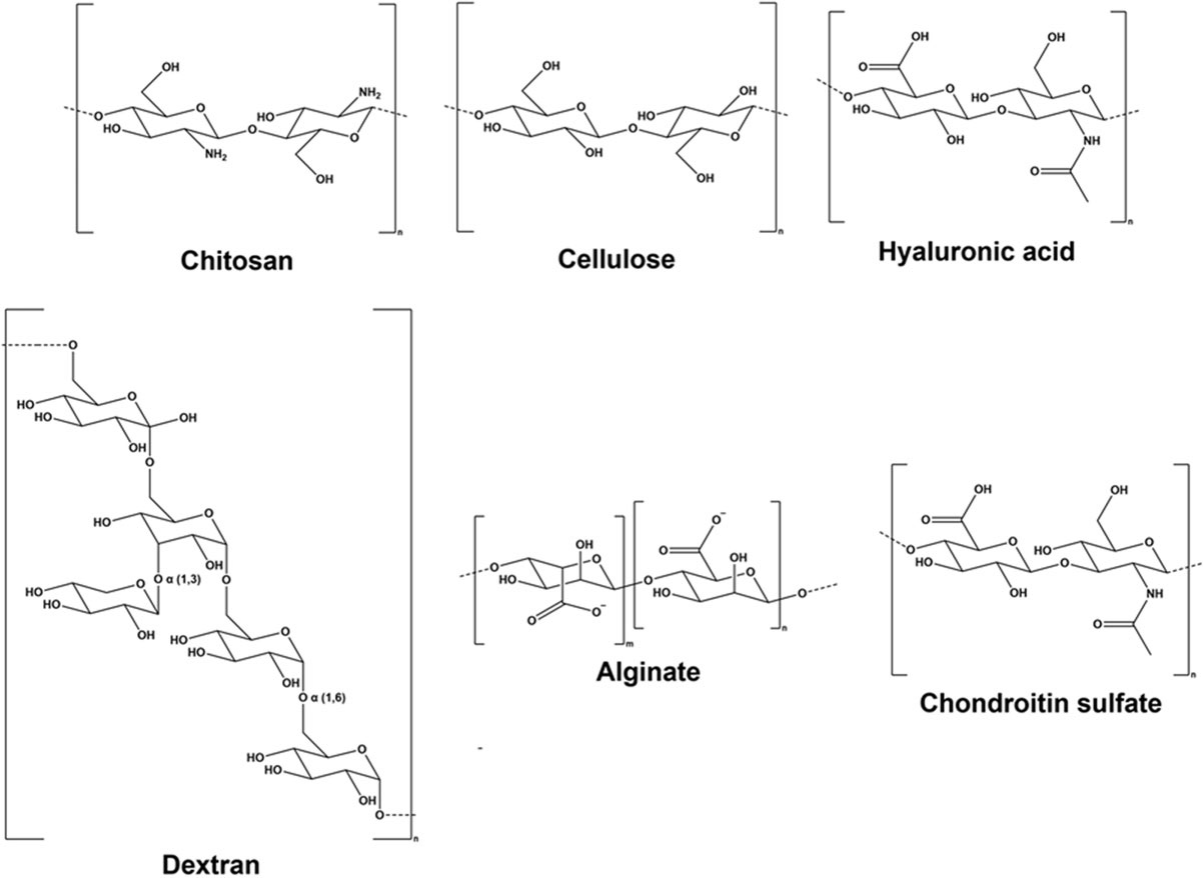
The chemical structures of various polysaccharides discussed in this review article.

Polysaccharides are long-chain polymeric carbohydrates abundantly found in food and are an essential component of living matter. Various biomedical and tissue engineering applications utilize polysaccharides because of their structural and biochemical similarity to the extracellular matrix. They are known to be biodegradable, biocompatible and easily tunable to achieve desired mechanical properties and tissue response [53, 54]. The following section details various polysaccharides and their use as hemostatic materials. Figure 2 depicts the chemical structures of the polysaccharides discussed in this review, while Table 1 summarizes and highlights key references of polysaccharide and polypeptide-based hemostatic materials discussed in this review.

**Table 1.** Summary of biopolymer-based hemostatic materials and their hemostasis time and blood loss in various *in vivo* animal models

Biopolymer	Form	Animal model	Treatment	Hemostasis time	Blood loss	References
Chitosan (CTS)	Particle	Rat Liver Injury Model	PECs w/10 wt% CTS	48 s		[67]
			PECs w/20 wt% CTS	67 s		
			PECs w/30 wt% CTS	82 s		
			Control: Gelatin sponge in powder form	121 s		
	Film	Rabbit Femoral Artery and Vein Model	CTS/Kaolin Composite	4.9 ± 1.6 min	3.5 ± 1.9 g	[68]
			Control: QuikClot Gauze	26.1 ± 22.8 min	25.1 ± 6.1 g	
			Control: Gauze	55.1 ± 13.8 min	31.1 ± 9.7 g	
		Rat Femoral Artery and Vein Model	CTS/Kaolin Composite	3.8 ± 1.4 min	1.3 ± 0.4 g	
			Control: QuikClot Gauze	8 ± 1.9 min	3.3 ± 0.7 g	
			Control: Gauze	>10 min	5.5 ± 1.4 g	
	Particle	Rat Tail Amputation Model	CTS Microspheres	214 ± 25 s		[69]
			CTS Microspheres w/thin layer of Calcium Alginate	161 ± 17 s		
			Control: Gauze	725 ± 21 s		
		Rat Liver Laceration Model	CTS Microspheres	107 ± 9 s		
			CTS Microspheres w/thin layer of Calcium Alginate	53 ± 10 s		
			Control: Gauze	238 ± 12 s		
Cellulose	Film	Rabbit Liver Model	Oxygen Regenerated Cellulose (ORC) film (oxidized 16 h)	192 ± 30 s	3.48 ± 0.82 g	[72]
			ORC film 44 h	178 ± 25 s	2.46 ± 0.66 g	
			ORC film 64 h	205 ± 40 s	3.06 ± 0.22 g	
			ORC film 88 h	233 ± 38 s	3.66 ± 0.54 g	
			Control: Gauze	300 ± 40 s	4.78 ± 0.53 g	
	Sponge	Mouse Tail Amputation Model	1 wt% Chitosan-Cellulose (CTS-Cel) Sponge	67 s	95 mg*	[80]
			Control: Gelatin Sponge	159 s	170 mg*	
			Control: Gauze	168 s	150 mg*	
			Control: No treatment	320*	210 mg*	
		Rat Liver Trauma Model	1 wt% CTS-Cel Sponge	89 s	400 mg*	
			Control: Gelatin Sponge	131 s	600 mg*	
			Control: Gauze	172 s	700 mg*	
			Control: No treatment	225 s*	900 mg*	
		Rat Leg Artery Model	1 wt% CTS-Cel Sponge	105 s		
			Control: Gelatin Sponge	372 s		
			Control: Gauze	486 s		
			Control: No treatment	840 s*		
Dextran	Sponge	NZ White Rabbit Marginal Vein Cut	Dextran-derived PDA Sponge	54 ± 6.0 s	0.11 ± 0.14 g	[84]
			Control: Celox	>420 s	2.65 ± 0.36 g	
		NZ White Rabbit Femoral Artery Cut	Dextran-derived PDA Sponge	<120 s	4.6 ± 0.55 g	
			Control: Celox	>180 s	0.1 ± 0.24 g	
	Hydrogel	Rat Liver Hemorrhaging Model	0.5 wt% Chitosan/Oxidized Dextran		53 mg	[87]
			1.0 wt% Chitosan/Oxidized Dextran		15 mg	
			0.5 wt% Chitosan		136 mg	
	Sponge	Rat Liver Hemorrhaging Model	Dextran-derived sponge	59.2 ± 3.8 s	0.52 g	[89]
			Control: Celox	108.7 ± 8.0 s	3.8 g	
			Control: Gauze	128 ± 8.0 s	4.7 g	
Alginate	Microspheres	Porcine Liver Punch	Thrombin-loaded Alginate-Calcium Microspheres	1.5 ± 1 min		[96]
			Thrombin-loaded Whole Blood	>10 min		
	Hydrogel	Rat Liver Hemorrhaging Model	Pept-1 (cell adhesive peptide) and Alginate		38 mg	[97]
			Control: No treatment		208 mg	
	Sponge	Mice Liver Hemorrhaging Model	Compressed Bi-Layer Alginate Sponge		18.3 mg	[95]
			Uncompressed Bi-Layer Alginate Sponge		232.4 mg	
			Control: TachoSil^®^		10.6 mg	
			Control: Surgicel^®^		19.0 mg	
HA	Hydrogel	Rat Liver Hemorrhaging Model	Self-crosslinking Gelatin Hydrogel		194.7 ± 140.5 mg	[100]
			HA/Gelatin Hydrogel		120.4 ± 149.5 mg	
			Control: Fibrin Glue		119.1 ± 77.6 mg	
			Control: No Treatment		233.8 ± 181.4 mg	
Chondroitin Sulfate (CS)	Powder	Porcine Liver Punch	CS, Collagen and Thrombin		0.46 g/min	[108]
			Gelatin-Thrombin matrix with smooth particles		0.14 g/min	
	Hydrogel	Mouse Liver Hemorrhage Model	CS–Serotonin Hydrogel		14.2 ± 0.8 mg	[109]
			Control: Chitosan-gelatin hemostatic agent		31.0 ± 7.7 mg	
			Control: No treatment		69.2 ± 11 mg	
Gelatin	Sponge	Dog spleen bleeding model	2-layer Gelatin sheet	104 ± 115 s		[116]
			Control: TachoSil^®^	277 ± 117 s		
	Sponge	NZ white rabbit ear bleeding model	Gelatin nanofiber sponge	112 ± 19 s	199 ± 32 mg	[148]
			Control: TachoSil^®^	133 ± 12 s	285 ± 34 mg	
			Gelatin nanofiber membrane	169 ± 20 s	318 ± 41 mg	
			Control: Gauze	266 ± 25 s	668 ± 108 mg	
		NZ white rabbit liver bleeding model	Gelatin nanofiber sponge	99 ± 11 s	131 ± 21 mg	
			Control: TachoSil^®^	128 ± 10 s	226 ± 27 mg	
			Gelatin nanofiber membrane	147 ± 13 s	238 ± 40 mg	
			Control: Gauze	237 ± 25 s	420 ± 91 mg	
	Cryogel	Rabbit liver defect hemorrhage	25 wt% Gelatin, 8 wt% Dopamine	83 s	1.2 g	[149]
			Control: Hemostatic sponge	222 s	4 g ± 1.5 g	
		Swine subclavian artery transection	25 wt% Gelatin, 8 wt% Dopamine	5.8 ± 1.4 min	193 ± 81 ml	
			Control: Gauze	25.4 ± 7.7 min	487 ± 142 ml	
	Particle	Rabbit Liver incision	Porous Gelatin microspheres (PGMs)	95.7 s		[118]
			Control: Chitosan Hemostasis Powder (CHP)	113 s		
			Control: Yunnan Baiyao	130 s		
		Rabbit Ear incision	Gelatin microspheres w/surgical gauze	45.3 s		
			Control: CHP	52.3 s		
			Control: Yunnan Baiyao	86.3 s		
Fibrin	Hydrogel	Human Vascular Surgery	3 ml fibrin sealant	3 min: 46.4% of patients; 4 min: 62.7%;		[121]
				5 min: 74.5%;		
				7 min: 100%		
			Control: Manual compression	3 min: 26.3% of patients; 4 min: 31.6%;		
				5 min: 49.1%;		
				10 min: 100%		
	Foam	Rabbit Liver Partial Resection	Fibrin Foam		50 ± 10 ml	[150]
			Control: No treatment		122 ± 11.5 ml	
	Film	Swine Spleen Incision	Fibrin patch	3 min: 86% success rate; 10 min: 100%		[124]
			Control: TachoSil^®^	3 min: 0% success rate; 10 min: 4%		
Keratin	Hydrogel	Rat Intracranial hemorrhage model (rebleeding)	0.2 U collagenase, then Keratin hydrogel		23.05 mm^3^ ± 9.67 mm^3^	[151]
			Control: 0.2 U collagenase		122.09 mm^3^ ± 25.25 mm^3^	
			0.4 U collagenase, then keratin hydrogel		42.09 mm^3^ ± 7.81 mm^3^	
			Control: 0.4 U collagenase		170.46 mm^3^ ± 25.25 mm^3^	
			0.6 U collagenase, then keratin hydrogel		60.87 mm^3^ ± 16.43 mm^3^	
			Control: 0.6 U collagenase		231.86 mm^3^ ± 32.28 mm^3^	
	Film	Rat liver puncture model	Full-length Keratin + PCL sheet	69 s	459 mg	[127]
			Rod domain Keratin + PCL sheet	60 s	368 mg	
			Alpha helical Keratin + PCL sheet	41 s	308 mg	
			PCL nanofiber sheet	155 s	671 mg	
			Control: Gauze	168 s	856 mg	
Silk Fibroin (SF)	Powder	Murine hepatic injury model	LMSF powder	120.6 ± 23.7 s	0.39 ± 0.04 g	[133]
			Control: No treatment	300 s	0.73 ± 0.04 g	
			Control: Arista^®^	110.5 ± 30.1 s	0.37 ± 0.05 g	
			Control: Surgicel^®^	102.8 ± 22.5 s	0.32 ± 0.04 g	
	Sponge	Rabbit liver trauma model	SF-PEG sponge	136.17 ± 62.27 s	2.16 ± 1.27 g	[134]
			Control: No treatment	557.75 ± 42.38 s	7.92 ± 0.8 g	
			Control: Gelatin sponge	249.83 ± 29.18 s	4.97 ± 1.44 g	
	3D scaffold	Rabbit ear artery hemorrhage model	TEMPO-oxidized cellulose nanofiber-5 wt% SF scaffolds with thrombin (TOCN-SF5-Th)	110 ± 5 s*		[135]
			TEMPO-oxidized cellulose nanofiber with thrombin (TOCN-Th)	225 ± 5 s*		
		Rat-tail amputation model	TOCN-SF5-Th	133 ± 14 s	0.59 ± 0.01 g	
			TOCN-Th	300 ± 6 s	2.3 ± 0.08 g	
			Control: Floseal^®^	120 ± 6 s	0.45 ± 0.08 g	
		Rat liver avulsion model	TOCN-SF5-Th	140 ± 5 s	0.84 ± 0.08 g	
			TOCN-Th	370 ± 6 s	2.0 ± 0.08 g	
			Control: Floseal^®^	140 ± 6 s	0.75 ± 0.08 g	
	3D scaffold	Rat tail truncation model	Tannic acid-SF with diclofenac potassium	160 ± 72 s	0.06 ± 0.03 g	[132]
			Control: Gauze	680 ± 60 s	0.58 ± 0.10 g	
Engineered Polypeptides	Spongy film	Murine liver trauma model	Fusion protein 96R	15.85 ± 1.21 s		[136]
			Control: RADA-16 lyophilized on gauze	14.44 ± 1.33 s		
			Control: No treatment	37.00 ± 1.75 s		
	Hydrogel	Rat liver trauma model	RADA16-1	<20 s		[137]
			Control: No treatment	>180 s		
	Hydrogel	Mice Liver Hemorrhaging Model	R-Gel-4	14 ± 4 s		[139]
			V-Gel-4	18 ± 2 s		
			Control: Fibrin glue	64 ± 10 s		
			Control: No treatment	170 ± 10 s		
	Bio-glue	Pig heart model	Cationic supercharged polypeptides (SUP) glue	75 ± 5 s*		[141]
			Control: Histoacryl^®^	110 ± 40 s*		
		Pig liver model	SUP glue	45 ± 10 s*		
			Control: Histoacryl^®^	100 ± 25 s*		
		Pig kidney model	SUP glue	30 ± 12 s*		
			Control: Histoacryl^®^	35 ± 10 s*		
	Hydrogel	Mouse liver bleeding model	Methacrylated elastin-like polypeptides		55.3 mg	[140]
			Control: No treatment		214.5 mg	
*L*-DOPA	High viscosity solution	Murine liver trauma model	PPDAC-PPDAL		0.36 ± 0.13 g	[142]
			Control: No treatment		1.72 ± 0.63 g	
	High viscosity solution	Hemorrhaging liver rat model	BPEDAC-BPEDAL		0.54 ± 0.11 g	[143]
			Control: No treatment		1.76 ± 0.56 g	

* Data extrapolated from figures.

### Chitosan-based materials

Chitin deacetylation is the primary source of chitosan synthesis. Strong alkali solutions are employed at both room and increased temperatures to remove *N*-acetyl groups during the deacetylation process. Chitosan is a heteropolymer chain with β (1 → 4) linked *D*-glucosamine and *N*-acetyl-*D*-glucosamine residues with <50% of *N*-acetyl-*D-*glucosamine units. It is a natural, non-immunogenic, biodegradable, non-toxic and mucoadhesive polysaccharide that has been investigated for use in a variety of biomedical applications [55, 56]. Chitosan is accessible for several recognized processing procedures due to its solubility in dilute acids and has been utilized to make films, gels and porous sponge-like scaffolds. Chitosan is insoluble in water at pH values >6.5 because it is a cationic polyelectrolyte with a pKa of 6.5. When coupled with a weak base-like glycerol phosphate, chitosan can generate an injectable thermogel [57]. At room temperature and physiological pH, this system stayed in solution. But when heated to physiological temperatures, it transforms into a gel, resulting in heat-induced gelation.

Due to its hemostatic and antibacterial qualities, chitosan has shown promise as a wound dressing material. The hemostasis activity is determined by a dynamic balance between anticoagulant and coagulating substances in the blood and blood vessels [58]. Recently, a chitin-based hemostatic agent was employed by Jorgensen *et al.* [59] for critical treatment during hemorrhage in an open wound. It has been reported that chitosan can shorten *in vitro* blood clotting time by 40% compared to blood alone [60]. Cationic chitosan contains coagulant characteristics, allowing red blood cells to have more active sites and activating platelets, which aids in developing a fibrin clot to stop bleeding [61]. Chitosan causes clotting due to the positive charge of the amino groups on the molecule, which interacts with the negative charge of red blood cell membranes [62]. A higher level of deacetylation results in a higher positive charge, which positively promotes coagulation [63]. The amount of coagulation has also been influenced by the type of chitosan employed, whether a solid or a solution. There was a dose-related clotting response, with increasing chitosan concentrations causing more coagulation [64]. Since 2003, chitosan-based hemostatic dressings have been utilized to treat injuries in military and civilian emergency response contexts, with encouraging results [65, 66]. In 27 of 34 cases in an emergency medical context, a chitosan dressing reduced hemorrhage within 3 min of administration. Twenty-one percent (7 of 34 incidents) of the failures were due to user error, which may have been avoided with better training and product design [65].

More recently researchers have combined the advantages of chitosan with other materials to enhance their hemostatic properties. For example, Chen *et al.* [67] prepared polyelectrolyte complexes (PECs) made up of chitosan oligosaccharide (COS) and carboxymethyl starch as absorbable hemostatic agents. They observed that PECs made up of 10 wt% COS improved hemostasis in a rat liver bleeding model, but the hemostasis efficacy reduced as the COS content increased. In a recent study, Elsabahy and Hamad [68] designed and evaluated chitosan/kaolin hemostatic dressings and utilized a surfactant to enhance the even distribution of kaolin throughout the chitosan fibers. They demonstrated their chitosan/kaolin dressings improved hemostasis and survival rates compared to QuikClot^®^ in both a rat and rabbit bleeding models. Wu *et al.* [69] prepared microspheres with a core made up of porous chitosan and a compact shell made up of alginate and demonstrated that this combinational microsphere promoted blood clot formation and was more effective as a hemostat compared to porous chitosan microspheres alone in both a rat tail amputation model and a rat liver bleeding model.

### Cellulose-based materials

**Figure 3.**
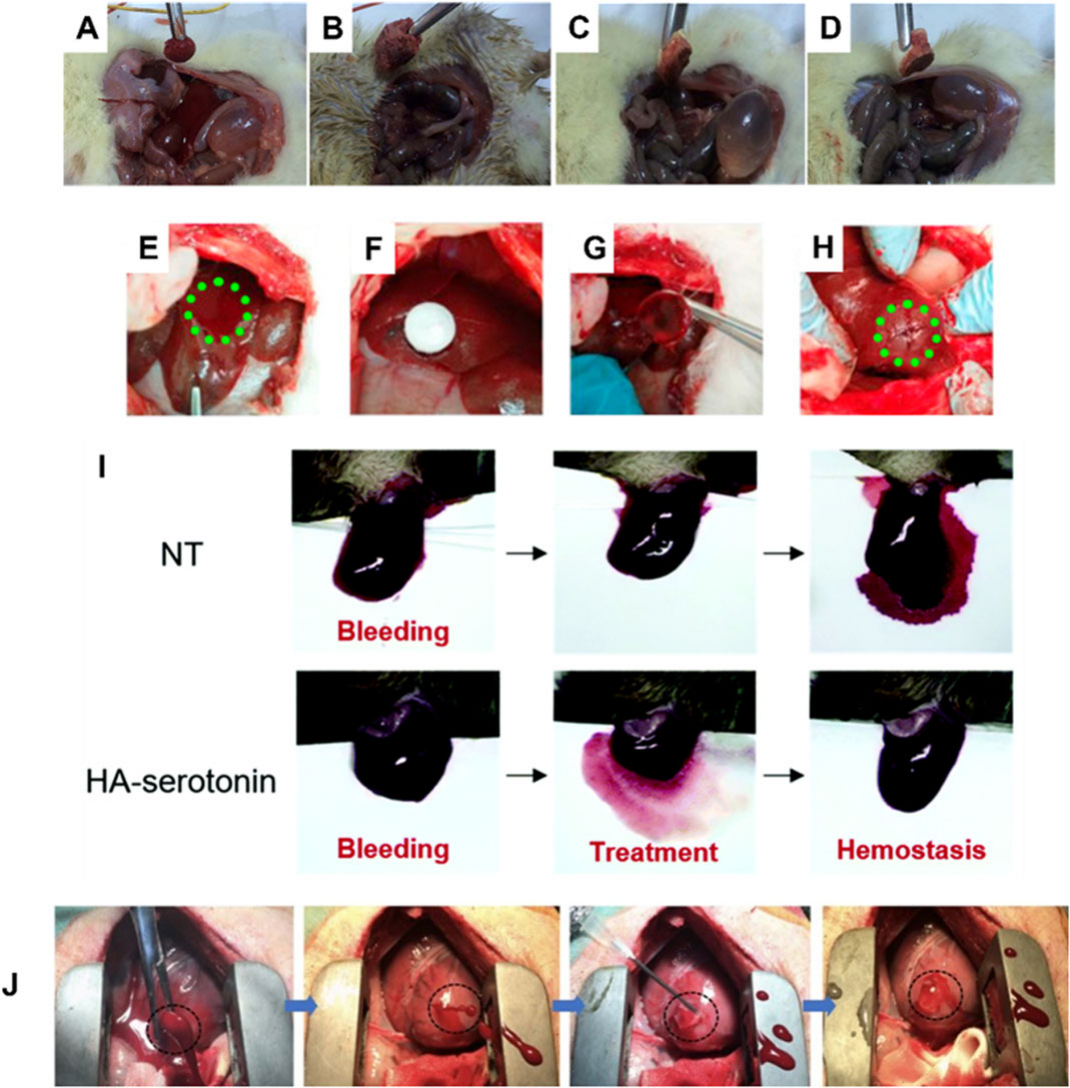
*In vivo* hemostasis studies using polysaccharide-based hemostats. (**A**) Standard gauze, (**B**) SF-coated bacterial cellulose/chitosan, (**C**) SF-coated Vit K/bacterial cellulose/chitosan, (**D**) SF-coated protamine sulfate/bacterial cellulose/chitosan applied to the bleeding site in a diabetic rat femoral artery model. Images adapted with permission from Karahaliloglu *et al.* [79], (**E**) creation of liver injury in the exposed left medial lobe of a New Zealand rabbit, (**F**) treatment of the injury site with aldehyde dextran (PDA) sponges. Hemostasis was maintained after removing the PDA sponge (**G**) and even after squeezing the wound (**H**). Images adapted with permission from Liu *et al.* [84]. (**I**) Liver hemorrhage model of factor VIII-deficient hemophilia mice with no treatment (NT), and HA-serotonin hemostatic adhesives. Image adapted with permission from An *et al.* [99]. (**J**) Six-millimeter cardiac puncture injury in pig hearts followed by treatment with methacrylated HA show rapid hemostasis and sealing following UV-induced polymerization. Image adapted with permission from Hong *et al.* [101].

Cellulose is a *D*-glucopyranose homopolysaccharide that contains a linear chain of *D*-glucose and is the most common organic polymer on Earth. Structurally, it is an essential part of the primary cell wall of green plants and many forms of algae, oomycetes and some bacteria biofilms [70, 71]. Due to its excellent biocompatibility, biodegradability, low costs and abundance, cellulose and its derivatives are commonly used as absorbable devices in wound dressings and hemostatic products [72–75]. Cellulose oxide (OC) is a cellulose derivative that is mainly investigated for hemostatic applications due to its ability to absorb liquids and traps platelets and erythrocytes quickly, which leads to an increase in the concentration of clotting factors, accelerates the clotting process when applied at the bleeding sites and facilitates the fibrin clots formation and block blood flow [76]. In the meantime, the carboxyl groups of OC triggers the coagulation cascade by self-activation of coagulation factor XII. It should be noted that, although OC has been extensively studied in hemostasis, its clinical application is significantly limited due to the acidic pH of various carboxyl groups [77]. To address such limitations, researchers have modified the structure of OC to improve its hemostatic applications. One of the popular methods to modify OC is adding other polysaccharides to enhance the hemostatic and improve the limitations of OC. For example, He *et al.* [78] coated chitosan on the surface of OC gauze and showed a significant improvement in OC hemostatic properties compared to traditional OC gauze. In another study, a bilayer wound dressing was prepared by Karahaliloğlu *et al.* [79] using chitosan and bacterial cellulose in the top layer, and (SF) on the bottom of the dressing. Their results indicated that applying such a platform on top of a bleeding area can quickly prevent bleeding by absorbing a lot of liquid from blood due owing to the bacterial cellulose and chitosan parts. Moreover, the top layer of SF can promptly cause platelet adhesion, which effectively showed a hemostatic effect *in vitro* and *in vivo* (Fig. 3A–D). In a more recent study, Fan *et al.* [80] prepared cellulose-based porous hemostatic sponges using surfactants and pore-forming agents. In comparison to traditional gauze and gelatin sponge, their cellulose-chitosan sponges showed improved hemostasis in several *in vivo* animal models, including mouse tail amputation model, as well as a rat liver trauma model and rat leg artery trauma model.

### Dextran-based materials

Dextran is another polysaccharide made up of a 1,6-linked *D*-glucopyranose residue. Dextran has many hydroxyl groups in its structure, facilitating chemical modification and the ability to add other functional groups. In addition, it has a high capacity to absorb water, making dextran a promising candidate for hemostatic application, specifically as a tissue adhesive agent [81]. Usually, sodium periodate (NaIO_4_) is used to oxidize the hydroxyl groups of dextran to change them to aldehyde groups. Amino groups of tissue proteins and other biomaterials can chemically crosslink with the aldehyde groups in dextran. This phenomenon produces strong adhesion to tissues, making them useful as tissue sealants [82]. However, the number and density of aldehyde groups in oxidized dextran are significant. It can affect local inflammation and systemic tissue toxicity when the oxidized dextran binds to tissues due to the tissue-material adhesion force [83]. Thus, the oxidation degree of dextran should be tuned based on its toxicity and adhesion. For example, Liu *et al.* [84] proposed a new aldehyde dextran sponge with improved water absorption and adhesive properties (Fig. 3E–H). The sponge showed quick blood absorption, powerful tissue adhesion and efficient hemostasis in a rabbit model. Furthermore, the aldehyde dextran sponge could facilitate wound blocking and cell aggregation and easily trigger blood coagulation without needing coagulation cascade activation.

In another study, Artzi *et al.* [85, 86] synthesized a sealant composed of star-shaped PEG-NH_2_ and dextran aldehyde with various molecular weights and degrees of aldehyde oxidation. Their results indicated that such materials could efficiently attach to tissue and prevent bleeding. Similarly, Du *et al.* [87] used modified chitosan and oxidized dextran to develop a novel hydrogel dressing, which demonstrated multifunctional activities to improve hemorrhagic and infected wound therapy in a rat hemorrhaging liver. Although oxidized dextran has been extensively investigated as sealants and tissue adhesives, the imine bond formation is an unstable equilibrium reaction in aqueous solutions. To address this limitation, Wang *et al*. [88] prepared a tissue glue made up of aldehyde dextran and gelatin, and incorporated 2-isocyanoethyl methacrylate into the backbone of dextran hydrogel to make the imine binding more stable. This modification improved the mechanical strength and stability of the hydrogels by significantly increasing the degree of crosslinking, which formed a dense intermolecular network. Most recently, Liu *et al.* [89] developed poly aldehyde dextran sponges loaded with montmorillonite powder (a natural silicate) for hemorrhage control. Their results suggest that their dextran-based hemostatic sponge exhibited good tissue adhesion, antibacterial activity against *Escherichia coli* and improved wound healing as well.

### Alginate-based materials

Alginate is a naturally derived polysaccharide from alga made up of *L*-glucuronic acid and *D*-mannuronic acid monomers. Alginate has been used for various biomedical applications due to its good biocompatibility, biodegradability and ability to quickly form a hydrogel when exposed to calcium or divalent ions [90–92]. However, when calcium alginate hydrogel comes in contact with blood, the calcium ions can exchange with sodium ions, making the gel lose. This exchange speeds up platelet aggregation and triggers the coagulation process, as the calcium ion works as a cofactor in the coagulation cascade [93]. In addition, calcium alginate can absorb high percentages of water, making it possible for calcium alginate gels to attach to the injury site and show an excellent hemostatic property. Shi *et al.* [94] synthesized carboxymethyl chitosan, sodium alginate and collagen composite microspheres and showed that their composites are biodegradable and facilitate platelet adherence, aggregation and activation *in vitro*. A systematic study by Singh Chandel *et al.* [95] evaluated the effect of sponge compression on bilayer alginate sponges prepared by lyophilization on hemostasis and antiadhesion. Their results indicated that their 100 µm compressed sponge demonstrated improved hemostasis in a mice liver bleeding model, while their 200 µm compressed sponge showed enhanced antiadhesion in a hepatectomy-induced adhesion model in rats.

In addition to fast gelation, alginate's easy drug loading capability has attracted researchers to investigate alginate in various fields. For example, Rong *et al.* [96] prepared thrombin-loaded calcium alginate microspheres prepared via emulsion/crosslinking technique and investigated the delivery of their hemostatic agent in an *in vivo* bleeding model. In another study, Zhai *et al.* [97] investigated a coassembly peptide and alginate system, which showed attractive cell adhesions and effective hemostasis. The proposed peptide-alginate hydrogel efficiently prevented bleeding in an *in vivo* mice model and reduced blood volume loss by 18% compared to the untreated group. Besides, the histology from a mouse full-thickness skin defect model indicated that the proposed peptide-alginate enhanced fibroblast migration to the injury site and facilitated wound healing.

### Hyaluronic acid-based materials

Hyaluronic acid (HA), commonly found throughout connective, epithelial and neural tissues, is another naturally derived polysaccharide made up of *D*-glucuronic acid and *N*-acetyl-*D*-glucosamine units. Due to its outstanding water maintenance and inherent swelling property, HA can facilitate cell adhesion and migration, making it one of the most suitable polysaccharides for wound healing as it facilities collagen production from wound surfaces via the fibroblast proliferation effect [98]. A study by An *et al.* [99] proposed serotonin-conjugated HA hydrogel systems as a new class of hemostatic adhesives. They determined that serotonin can boost hemostasis, and their hydrogels showed improved hemostatic ability in regular and hemophilic lesions in a rat model compared to commercially available fibrinolytic, making them good candidates for hemorrhage control (Fig. 3I) [99].

In another study, Luo *et al.* [100] synthesized two types of injectable self-crosslinking gels using gelatin and HA, and investigated them for use in hemorrhage control. Their HA-gelatin hydrogels had excellent stability, low cytotoxicity, beneficial burst strength and remarkable hemostatic ability compared to commercial fibrin glue. Hong *et al.* [101] developed methacrylated HA hemostatic hydrogels as a strongly adhesive for arterial and cardiac injury applications (Fig. 3J). The proposed material can rapidly gel upon UV light irradiation and form hydrogels that can adhere to and seal the bleeding arteries and heart walls, withstanding up to 290 mmHg blood pressure. Meanwhile, their results indicated that the treated heart pig model could survive for several days after incision, suggesting that their hemostatic hydrogel would be suitable for use as a traumatic wound sealant.

### Chondroitin sulfate-based materials

Chondroitin sulfate (CS) is the most prevalent glycosaminoglycan in the body, composed of repeating units of glucuronic acid and galactosamine. It is present in various structural proteoglycans in multiple tissues, including skin, cartilage, tendons, heart valves and the central nervous system. CS is a biodegradable, anti-inflammatory, antioxidant, anticancer, anticoagulant and anti-thrombogenic anionic heteropolysaccharide [102]. CS has several critical functions, including resistance to compression forces and activation of crucial pathways involved in vascular healing. CS can regulate cellular processes, such as cell motility and receptor binding. It also has anti-apoptotic and antioxidant properties and plays a crucial role in immunological responses, such as growth factor activity modulation and leukocyte recruitment. Furthermore, CS-based hydrogels have a high wound healing potential, and cellular biological activity [103, 104].

Moreover, due to its affinity for CD 44 receptors and glycosylation enzymes on the surface of tumor cells and intracellular organelles, it also inherits the potential to target active and subcellular targets [105, 106]. CS degrades in the presence of physiological stimuli; it could be a viable biomaterial for the delivery of biopharmaceuticals and stimuli-sensitive delivery systems like tumor-targeted delivery [107]. For individuals with a high risk of bleeding, CS has the potential to be a successful treatment [105, 106].

Slezak *et al.* [108] developed an active powdered hemostatic product of swine collagen, bovine CS and plasma-derived human thrombin that can be applied directly to the bleeding site. Compared to flowable agents, powdered hemostatic agents can be applied directly over large surface areas when the source of bleeding is unknown. However, their efficacy is limited to little bleeds. Zhang *et al.* [109] developed a highly biocompatible serotonin-conjugated CS hydrogel and demonstrated improved hemostasis and wound healing abilities in an *in vivo* model.

## Polypeptide materials for hemostasis

Polypeptides are continuous, unbranched chains of amino acids linked by peptide bonds that connect amine and carboxyl groups of adjacent amino acids to make an amide bond. Polypeptides are crucial building blocks for designing biomaterials because of their capacity to form well-defined secondary structures (i.e. -helix and -sheets). These secondary structures play an essential role in polypeptide chain self-assembly, resulting in unique supramolecular structures [110]. Furthermore, they can have a range of reactive functional groups (carboxylic acids, hydroxyl, amino and thiol groups) on their side chains, which can be easily chemically modified [111]. The most significant limitation of peptide-based polymers is their limited number of building blocks. They are limited to the 20 natural amino acids compared to synthetic polymers, which can be made from many monomers [112]. On the other hand, these biomaterials are in high demand due to several advantages. Short peptide motifs like RGD, which are abundant ligands for cell receptors and regulate various cell activities, such as attachment and spreading, may be attached to or embedded more easily in polypeptide materials than synthetic materials [113].

Furthermore, because many peptide-based polymers are readily degradable by the body, they are good candidates as biomaterials. Moreover, peptide self-assembly has recently attracted scientific attention as a potential method for producing functional biomaterials [114]. Due to their adhesive properties, polypeptide biomaterials have also been reported as hemostatic materials, tissue adhesives and wound healing applications. This section highlights the advantageous properties of various adhesive materials derived from polypeptide compounds in hemostasis.

### Gelatin-based materials

**Figure 4.**
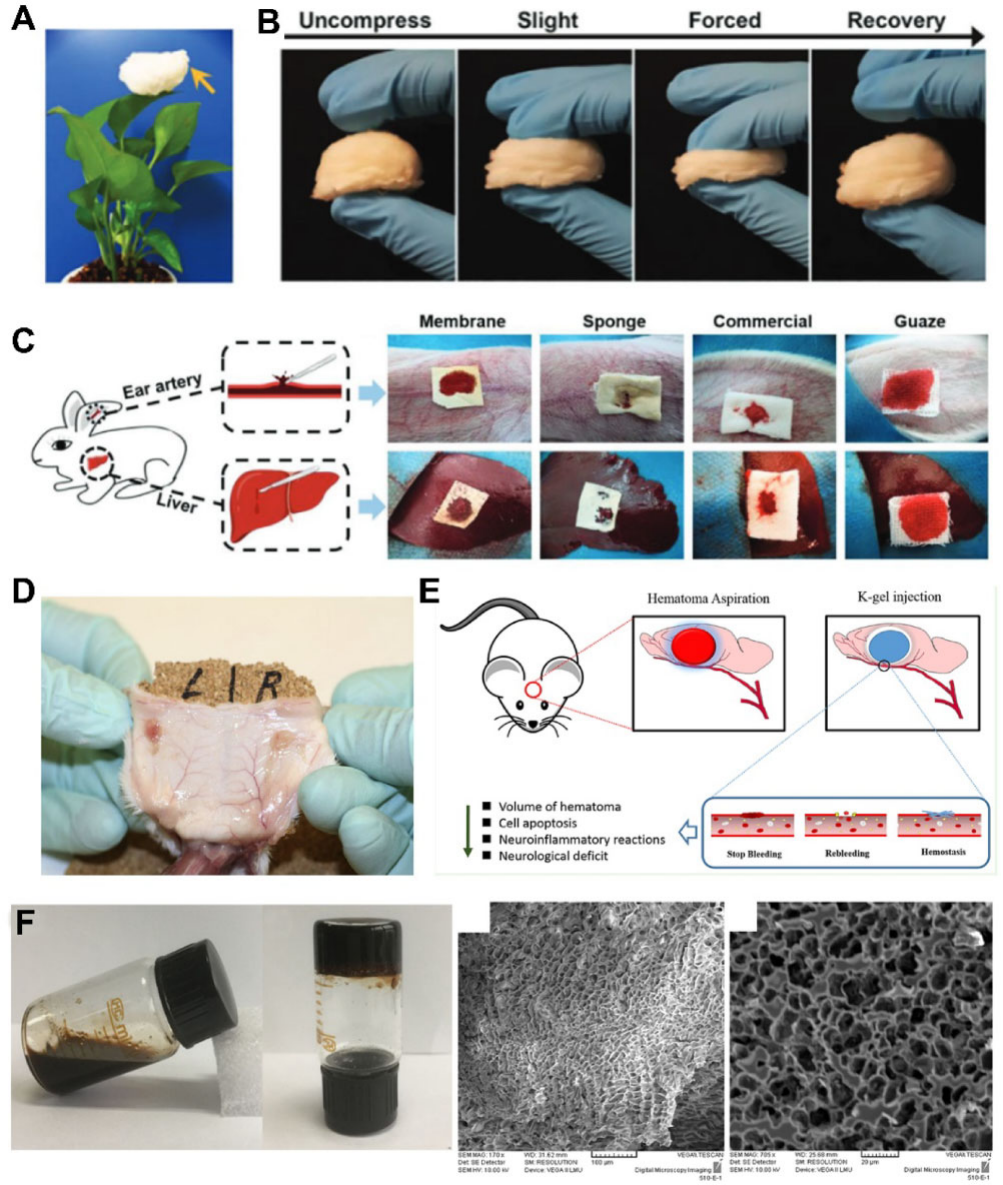
(**A**) Ultralight gelatin nanofiber sponge. (**B**) Compressibility and recovery properties of the gelatin nanofiber sponge. (**C**) *In vivo* rabbit model evaluation of the hemostatic capacity of the ultralight gelatin sponge in comparison to sponge, gauze and commercial product. Image adapted with permission from Xie *et al.* [117]. (**D**) Fibrin pad (subcutaneously implanted; on the left), shows good vascularization after implantation day 14 compared to the control on the right. Image adapted with permission from Harmon *et al.* [123]. (**E**) Human hair hemostatic keratin hydrogels for the treatment of intracerebral hemorrhage were tested in the rabbit model. (**F**) Keratin solution and hydrogel showing gelation properties, scanning electron microscope (SEM) images of the gels. Images adapted from He *et al.* [128].

Gelatin is a denatured form of collagen and is known to possess optimal properties as a material for various biomedical applications, including hemorrhage control [115]. The hemostatic effect of gelatin is attributed to the swelling of the biopolymer upon contact with blood as well as activation and aggregation of platelets which in turn accelerates blood coagulation [116]. However, gelatin suffers from poor mechanical properties at physiological temperatures. Therefore, it is typically combined with other materials, such as levodopa (*L*-DOPA), or nanosilicates, such as Laponite. Xie *et al.* [117] developed an electrospinning method to create an ultralight nanofiber gelatin sponge with a porous topology and large surface area (Fig. 4A–C). According to the researchers, the nanofiber sponge, made up of gelatin aggregates, activated many platelets, promoting platelet embolism and escalated coagulation pathways. Furthermore, *in vivo* studies demonstrated the ability of these gelatin sponges to generate stable blood clots in a short amount of time and exhibited negligible blood loss. Li *et al.* [118] prepared porous gelatin microspheres using a water-in-oil emulsion method, followed by glutaraldehyde crosslinking, and lyophilizing after freezing in liquid nitrogen. Their experiments demonstrated the use of their microspheres as hemostatic agents in both *in vitro* and *in vivo* studies compared to hemostatic powder controls.

### Fibrin-based materials

After sustaining an injury, the blood coagulation pathway gets activated, and a fibrin clot forms to prevent bleeding. Fibrin generates a fibrous matrix with important biomechanical characteristics, such as extensibility, stiffness, resistance to rupture, and optimal elastic and viscous characteristics, making it suitable for hemostatic applications [119, 120]. Fibrin sealants are typically composed of human-derived fibrinogen and thrombin, which help to increase coagulation factors and activate blood clotting [24, 121, 122]. To prevent blood loss, the sealants also create a barrier for sealing. Several commercially available fibrin sealants are FDA approved for use in surgical procedures to achieve hemostasis. These include Tisseel and Artiss from Baxter, Evicel and Vistaseal from Johnson & Johnson, and Vitagel from Orthovita [31]. Harmon *et al.* [123] developed a fibrin pad for hemostasis for surgical interventions with good healing properties (Fig. 4D). Their fibrin pad, composed of fibrinogen and thrombin-loaded matrix component, served as a hemostatic patch by delivering fibrin to the injury site. In another study, Matonick and Hammond [124] compared two FDA-approved fibrin-based sealant patches, EVAREST™ and TachoSil^®^, in a heparinized swine spleen incision model. Their results indicated that EVARREST™ outperformed TachoSil^®^ in both hemostasis success rates and adhesion to wound tissue.

### Keratin-based materials

Keratin is a family of proteins containing cysteine amino acids rich in sulfur. It is a fibrous biomaterial commonly found in hair, nails, wool, etc. Keratins are categorized as either hard or soft keratins, where the sulfur crosslinking contributes to the hardness of the polymer. They can also be classified as alpha and beta keratins, depending on the source of the keratin and the tertiary structure of the polymer (alpha helices or beta sheets) [125]. Keratin biomaterials are often used for drug delivery, bone regeneration and wound dressing. However, they recently attracted attention as hemostatic materials because they reduce clotting times and blood loss [126]. In addition, they cause polymerization of fibrinogen into fibrin which also contributes to hemostasis. In a comparative study by Wang *et al.* [127], they observed that the α-helical segment contents of keratin are directionally proportional to its hemostatic activity, while the Tyr, Phe and Gln amino acids at the N-termini of the α-helices in keratins play an important role in fibrin polymerization. He *et al.* [128] fabricated keratin-based hydrogels extracted from human hair, named K-gels (Fig. 4E and F). Their gels showed excellent biocompatibility, promising hemostatic efficacy and demonstrated the potential to relieve brain damage *in vivo* by preventing bleeding that may happen postoperatively.

### Silk-based materials

**Figure 5.**
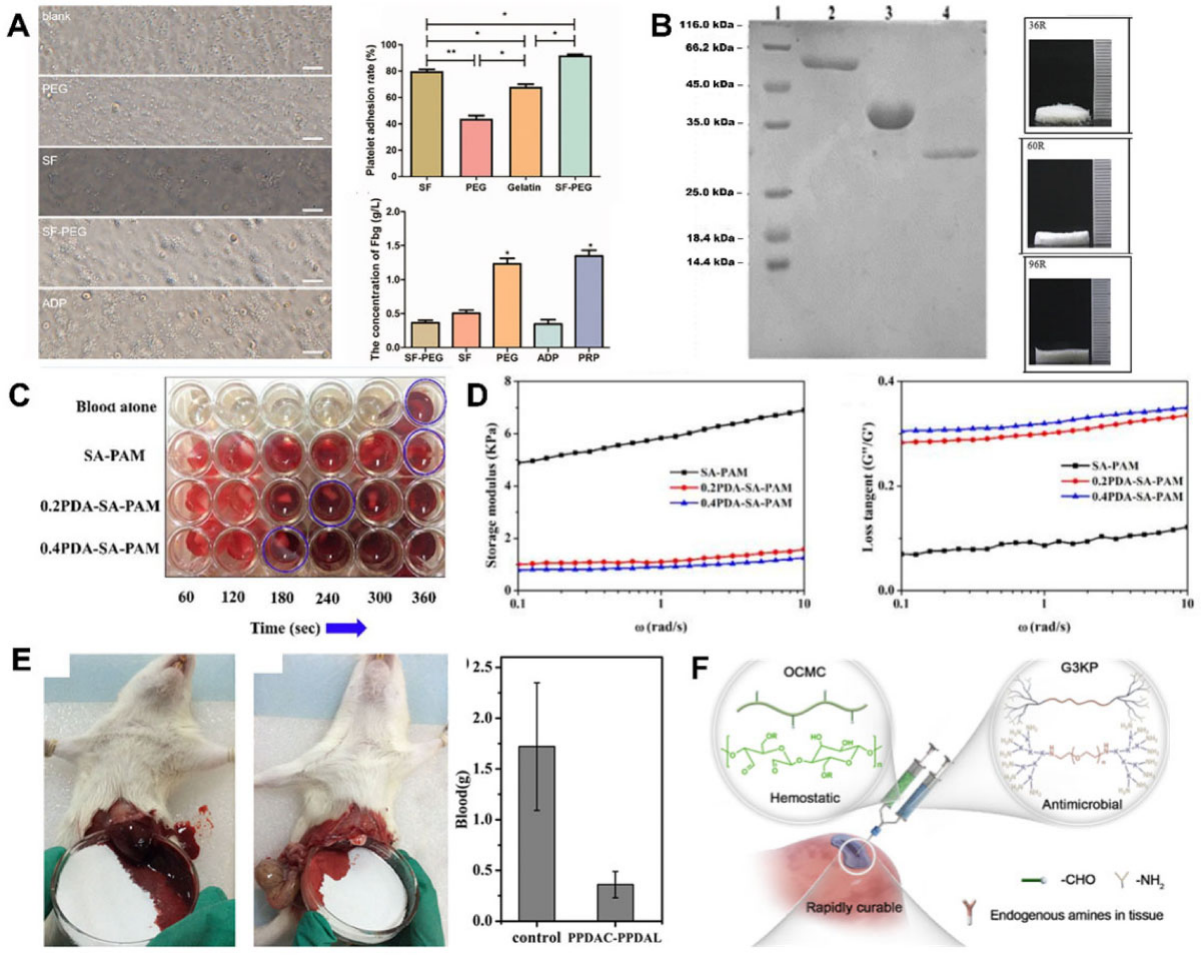
(**A**) Platelet adhesion and fibrinogen concentration in response to silk fibroin and SF-PEG sponges. Image adapted from Wei e*t al.* [134]. (**B**) SDS-PAGE showing three hemostatic peptides and their sponges after purification. Image adapted from Yang *et al.* [136]. (**C**) PDA–sodium alginate–polyacrylamide (PDA–SA–PAM) hydrogel network showing accelerated clotting time [144]. (**D**) The addition of polyDOPA to SA–PAM structure improves rheological properties drastically. Images adapted from Suneetha *et al.* [144]. (**E**) Hemostatic capacity of the triblock DOPA peptide. Image adapted from Lu *et al.* [142]. (**F**) Hemostatic OCMC and antimicrobial G3KP polysaccharide-peptide dendrimers for rapid curing tissue adhesive-hemostat development. Image adapted from Zhu *et al.* [147].

SF is most commonly obtained from *Bombyx mori* cocoons [129] and was approved for sutures and surgical materials more than two decades ago by the FDA [130]. SF is made up of a heavy and a light chain bound together with disulfide bonds and can assume either an α-helical amorphous structure or more crystalline β sheets [131]. It can be processed into mats, foams, fibers or hydrogels, although it requires longer and more complicated processes than previously mentioned biopolymers. As a result of this, SF by itself is not of great interest for hemostasis and is often combined with other materials. Zhu *et al.* [132] demonstrated improved hemostasis and biocompatibility of SF hydrogels with tannic acid and diclofenac potassium in *in vitro* studies and a rat tail truncation model. Lei *et al.* [133], however, modified SF by hydrolysis to obtain low molecular weight SF (LMSF) and showed that LMSF activates the intrinsic pathway and decreases bleeding time and blood loss. On the other hand, Wei *et al.* [134] developed an SF-polyethylene (SF-PE) sponge gellable in aqueous physiological environments. While SF showed superior platelet adhesive properties, SF-PE sponges shortened hemostatic time and decreased blood loss compared to SF alone or gelatin commercial sponges (Fig. 5A). In another study, Shefa *et al.* [135] combined SF with nanocellulose and enhanced the hemostatic properties by loading sponges with thrombin. The addition of SF improved the sponge’s biocompatibility and blood absorption capacity, while thrombin enhanced its hemostasis.

### Engineered polypeptides

In recent years, engineered self-assembling peptide materials have evoked interest in hemostatic biomaterials research. Yang *et al.* [136] developed RADA-16-based biomolecules with hemostatic effects. It was reported that the peptide can quickly self-assemble to form a layer of fibers that acts as a barrier to block hemorrhage in <15 s [137]. It has amphipathic properties that prevent bleeding wounds in the brain, liver, skin and spinal cord (Fig. 5B) [138]. Teng *et al.* [139] engineered glycopolypeptides using a poly-*L*-lysine backbone grafted with glucose and catechol groups crosslinked to form covalent hydrogels. These gels exhibited strong tissue adhesion and showed efficient hemostatic properties. In addition, they reported that their gels regenerated dermis and epidermis tissues holding potential as high-performance hemostats and wound dressings. Guo *et al.* [140] synthesized methacrylated lysine-rich elastin-like peptides (ELP-MA) with lower transition temperature and the ability to photocrosslink. Furthermore, their ELP-MA showed improved hemostasis in a mice liver bleeding model and functioned as elastic adhesives. Ma *et al.* [141] designed a family of supercharged polypeptide-based adhesives demonstrating strong adhesion to soft tissues, which outperformed some commercially available adhesive products. The supramolecular interactions between cationic supercharged polypeptides and anionic aromatic surfactant with lysine resulted in strong adhesion to tissues. In addition, their bio-glue showed robust *in vitro* and *in vivo* performance for hemostasis application and wound healing compared to surgical wound closures.

### Mussel-inspired l-3,4-dihydroxyphenylalanine (*L*-DOPA) materials

When exposed to high humidity, marine mussels show strong adherence to surfaces [142]. These adhesive properties can be attributed to the amino-peptide compound *L*-DOPA containing catechol functional groups capable of forming strong covalent and non-covalent interactions and lysine near the interface [143]. As a result, the *L*-DOPA motif has been incorporated into many polymeric backbones to develop novel materials with adhesive and hemostatic properties. Furthermore, the amino and phenolic hydroxyl groups of DOPA also activate the coagulation system. Suneetha *et al.* [144] developed polydopamine–sodium alginate–polyacrylamide (PDA–SA–PAM) hydrogel networks with high porosity. The polydopamine chains in this hydrogel significantly improved the scaffold’s mechanical properties and contributed to faster coagulation (Fig. 5C and D).

Lu *et al.* [143] investigated a series of novel biomedical adhesive gels derived from *L*-DOPA. Their polymers demonstrated biocompatibility and good biodegradability as well as thermoresponsiveness. Furthermore, these polymers excelled in mechanical properties and *in vitro* adhesion tests without showing cytotoxicity. They also performed *in vivo* antibleeding studies where their polypeptide materials demonstrated superior hemostatic properties. In another study, Lu *et al.* [142] developed thermoresponsive polypeptide-pluronic-polypeptide triblock copolymers. These triblock copolymers had different functional side groups showing good biodegradability and biocompatibility and excellent hemostatic properties. The hemostatic properties were due to rapid polymerization and solidification of the polymer (Fig. 5E). Furthermore, they demonstrated that these glues had excellent wet adhesive properties. These results demonstrate the potential for these mussel-inspired materials to act as high-performance hemostatic materials.

## Polysaccharide and polypeptide composite materials for hemostasis

Several studies have combined polysaccharides and polypeptides to mimic the natural extracellular matrix structure. These combinations have the advantage of adding the properties of both materials to improve their adhesiveness. For example, poly-lysine, which occurs naturally, is biodegradable and non-toxic. It also shows tissue adhesive properties stemming from the ionic interaction between the polymer and target tissues. Nie *et al.* [145] combined chitosan and poly-lysine, which showed high functionality at adhesion sites, excellent hemostatic properties when applied to a rat liver defect and proved to be a promising novel hemostatic material also functioning as a tissue sealant. Lu *et al.* [146] also took advantage of the properties of both polysaccharides and polypeptides by combining chitosan and the marine mussel-inspired *L*-DOPA peptide. They generated a series of chitosan-polypeptide polymers having different functional groups. These materials exhibited high adhesion strength and showed good hemostatic performance. Zhu *et al.* [147] described a fast and high-strength bioadhesive hydrogel based on polysaccharide and peptide dendrimers named OCMC/G3KP (Fig. 5F). Their hydrogels demonstrated excellent hemostatic properties with a 5-fold increase in adhesion strength compared to CoSeal, a commercially available bioadhesive.

## Various forms of hemostat devices from biopolymers

Hemostasis is a biological process ensured by closely controlled coagulation pathways, including coordinated enzyme activities. In cases of extreme blood loss, the body's natural hemostasis mechanism cannot control bleeding; hence adjuvant methods for bleeding control are required. Generally, these hemostatic devices should be readily available, easily transportable, storable and applicable even in austere environments. Understanding the natural hemostasis process is critical in developing viable hemostat devices that mimic or stimulate the natural process [30]. During World War II, biopolymer-based hemostat devices received significant attention. They were immediately accepted by the medical community and were shown to be entirely safe for human use [152]. The ability of hemostatic materials to limit blood loss and biofilm formation is crucial to their clinical value. Materials including hydrogels, sponges, woven and non-woven fiber sheets, and powders have been widely used [153–155].

The primary objective of hemorrhage treatment is to stop bleeding and restore blood volume that is circulating within the body [156]. The likelihood of survival could very well be determined by the severity of the bleeding. Patients experiencing moderate hypotension due to bleeding may benefit from delayed massive fluid restoration until they reach a medical treatment facility that provides definitive care [157]. Generally, hemorrhage or bleeding can be categorized into three classes: arterial bleeding, venous bleeding and capillary bleeding [158]. Arterial bleeding occurs when there is a wound or incision to a major artery, resulting in rapid reduction of blood volume. Venous bleeding occurs when there is a wound or incision to a vein. Like arterial bleeding, blood loss from venous bleeding can be substantial and occur quickly without intervention. Finally, capillary bleeding occurs due to an incision or wound to capillaries with minimal blood loss which can be easily controlled. The key first aid treatment for all these types of bleeding is direct pressure over the wound followed by using different hemostasis agents to stop the bleeding [159].

In addition, hemorrhages can also be categorized into four classes based on various parameters, such as, blood loss, pulse rate, blood pressure, respiratory rate, urine output and central nervous system symptoms. The primary observation of the parameters, as seen in Table 2, may be helpful to medical personnel in providing the most appropriate treatment and medication to stop bleeding and increase the patient's chance of survival [156].

**Table 2.** Classification of hemorrhage

Parameter	Class			
	I	II	III	IV
Blood loss (ml)	<750	750–1500	1500–2000	>2000
Blood loss (%)	<15%	15–30%	30–40%	>40%
Pulse rate (beats/min)	<100	>100	>120	>140
Blood pressure	Normal	Decreased	Decreased	Decreased
Respiratory rate (breaths/min)	14–20	20–30	30–40	>35
Urine output (ml/hour)	>30	20–30	5–15	Negligible
CNS symptoms	Normal	Anxious	Confused	Lethargic

The first biological-based hemostat devices, also called first-generation hemostat devices, were single-component products using thrombin, fibrin, collagen and cellulose. Among them, thrombin and fibrin were the first materials to be surveyed using the pioneering study developed by Hong and Loughlin [160] The most significant developments in the use of naturally occurring and synthetic polymers as hemostatic devices were recently reviewed by di Lena [161] While, Biswal [162] have surveyed the applications of biopolymers for tissue engineering, especially in hemostatic devices. Besides, functional tuning associated with specific hemostatic components (thrombin or fibrin) is mainly produced to achieve better hemostatic performance, particularly in malfunctioning the natural coagulation process [163].

Researchers have demonstrated a high interest in naturally generated biomaterials for various biomedical applications. Bose *et al.* [164] describe that biopolymers made up of proteins and cellulose have been used to stop bleeding for a long time. The primary goal of biopolymer hemostat devices is to reduce surgical complications by providing easy handling and optimal hemostatic capability [152, 165]. Furthermore, they should be non-stick and possess enough mechanical strength to withstand the bleeding pressure [166, 116]. The following section highlights various forms of biopolymer-based hemostatic devices.

### Powder and particles

Powders and particles are a beneficial and practical form of hemostatic materials. They can halt blood loss rapidly, can be easily applied, have a long shelf life, can be stored in various temperature settings, and pose no risk of disease transmission to patients [167]. Generally, microparticulate hemostats, such as ChitoHem [168], Arista [169] and Quickclot [24], were developed because of their superior hemostatic activity and simplicity. Recently, some researchers have proposed nanoparticles and beads as hemostatic devices for rapid blood coagulation in several *in vivo* studies [170–172].

In terms of hemostatic material development, many materials show positive results due to their biocompatibility and simplicity [173]. Oxidized regenerated cellulose (ORC) is a natural-based polymer derived from chemical modification of cellulose. Due to its biocompatibility, biodegradability, low toxicity and low cost, among other characteristics, it has been widely used for many biomedical applications [163]. Hutchinson *et al.* [76] described that ORC could be utilized as a hemostat due to its exceptional behavior in stopping bleeding. Two different ORC powders with sodium or potassium were tested *in vitro* for bactericidal action by Basagaoglu Demirekin *et al*. [163]. Furthermore, the chemical characterization of ORC was carried out using the textile form of the compound and regenerated cellulose. However, due to the differences in material form, they were unable to be used in *in vivo* research due to safety concerns. The study aimed to determine whether they were effective as a hemostatic substance. These materials had bactericidal action because of the acidic features of ORC and biocompatibility and efficiently halt bleeding.

A study on QuikClot (a zeolite-based granular hemostatic substance) was undertaken by Pusateri *et al*. [9] in a pig liver laceration model to determine its effectiveness. Jegatheeswaran *et al.* [174] reported a swine liver laceration model was used to test the feasibility of a chitosan-based powder hemostat. They found no indication of an inflammatory response or heat damage but did find some evidence of sinusoidal congestion. Yang *et al.* [173] developed polysaccharide-based hemostatic materials with antimicrobial and healing properties. Sakoda *et al.* [175] presented a hydrogel hemostat consisting of hyaluronan (HA) coupled with inorganic polyphosphate as a treatment option for hemorrhage control. Aldehyde-modified HA and hydrazide-modified HA coupled with PolyP quickly generated HAX-PolyP. In a mouse liver bleeding model, HAX-PolyP had the same hemostatic effect as fibrin glue in accelerating the coagulation rate of human plasma *ex vivo*.

Panwar *et al.* [176] developed a biodegradable and biocompatible hemostatic device by conjugating carboxymethyl moiety to starch (CM-starch). They altered it with calcium ions (CaCM-starch) to generate free-flowing microparticles, which were injected into wounds. *In vitro* and *in vivo* research on hemostatic efficacy indicated CaCM-starch as a promising candidate for further clinical evaluation as a topical hemostat. Zhu *et al.* [177] reported a crosslinked starch microparticle to improve hemostasis during irregular surgical procedures.

### Sponges and foams

Sponges are highly absorbent porous structures holding great promise in tissue engineering and regeneration [178–182]. Especially, their unique swelling/shrinkage properties render them desirable for hemostatic applications, where they can fill the wound cavity and absorb lots of blood [183]. Various studies have reported processing synthetic and naturally derived materials into sponges for hemorrhage control [164, 184–185]. Naturally derived materials have been increasingly attractive compared to synthetic materials due to their better biocompatibility, biodegradability and low/no toxicity [186, 187]. A great range of biopolymers and derivatives have been processed into hemostatic sponges, such as gelatin [148, 188], chitosan [185, 189, 190], cellulose [154, 183] and alginate [191].

**Figure 6.**
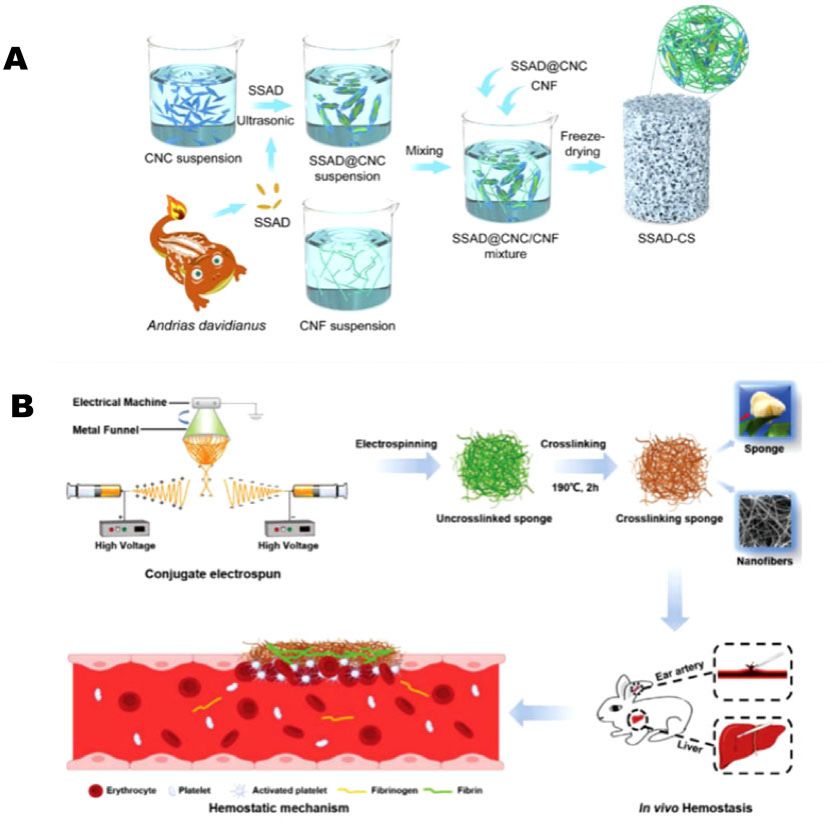
Examples of biopolymer-based hemostatic sponges. (**A**) SSAD (skin secretion of *Andrias davidianus*)-enabled cellulose hemostatic sponges. Image adapted with permission from Zheng *et al.* [183] Copyright 2021, Elsevier. (**B**) Demonstration of fabrication, blood clotting and the hemostatic mechanism of 3D gelatin nanofiber-based sponges. Image adapted with permission from Xie *et al.* [148] Copyright 2021, Wiley.

Zheng *et al.* [183] used skin secretion of *Andrias davidianus* (Chinese giant salamander), combined with cellulose nanocrystals and cellulose nanofibers to fabricate injectable hemostatic sponges that were shape-recoverable, elastic and had a high blood absorption ratio (Fig. 6A). *In vitro* performance of as-prepared sponges was assessed, with the results showing significant volumetric expansion (>11.54) and high water absorption ratios (up to 6276 ± 398%). The sponges outperformed the hemostatic effects of pure cellulose and gelatin sponges *in vivo* [183]. In another study, porous and hydrophilic composite sponges comprising cellulose and chitosan were fabricated with incredible mechanical resilience [184]. Incorporating chitosan in the sponges resulted in antibacterial efficacy against both gram-positive and gram-negative bacteria. These composite sponges with high water absorption capability could better facilitate coagulation compared to commercial gauze and gelatin sponges, *in vivo*, and showed rapid hemostasis in the rat liver bleeding model.

SF has attracted much attention recently because of its tunable mechanical properties and great processibility [192–195]. As a result, SF-PEG sponges have been explored [134]. The authors proposed that SF itself could impact the hemostatic process by activating platelet aggregation and adhesion. Mixing with PEG could further accelerate the hemostatic process because PEG-SF structural networks serve as a physical barrier to the bleeding area [134]. Another example was reported by Teuschl *et al.* [196] where they used SF as a carrier for delivering fibrinogen and thrombin to control blood loss. The silk sponges showed excellent mechanical robustness as the physical barrier. The delivery of these coagulation proteins facilitated the clotting of hemorrhaging wounds under dysregulated thrombin and fibrinogen levels and illustrated the promising role of SF as the carrier for various bioactive agents for biomedical applications [196].

Gelatin has been adopted for hemostatic applications due to its ability to cause platelet aggregation [149]. Xie *et al.* [148] reported a conjugate electrospinning method to engineer 3D gelatin-based hemostatic sponges (Fig. 6B). The resulting sponges have interconnected nanofiber networks, porous structure and a high surface area, with excellent hemostatic capability. *In vitro* assessments of blood coagulation were performed and revealed that the 3D microstructured nanofibers could trap blood to shorten the bleeding time. Compared to traditional gauze and commercial hemostatic agents and membranes, the proposed sponges demonstrated great *in vitro* blood-clotting ability with a blood-clotting index of 21.9 ± 4.4% and blood-clotting time of about 180 ± 15 s. Compared to the commercial gelatin sponge (133 ± 12 s), the membrane (169 ± 20 s) and gauze (266 ± 25 s), the sponge showed a faster clotting efficiency of 112 ± 19 s in a rabbit ear artery injury model. The mechanism can result from a great surface area and highly porous structure inside the sponges, and these factors benefit the quick absorbance of wound exudes, blood cells and platelets [148].

Regarding clinical translation, sponges for the control of bleeding during surgery are commercially available and accepted for medical usage [187]. Pfizer has marketed Gelfoam^®^, a hemostatic sponge made from porcine skin gelatin and used as an FDA-approved medical device for bleeding surfaces [197]. Similarly, Spongostan™ gelatin sponge features resorbable, malleable and water-insoluble properties, which prevent blood loss from the wound cavity [198]. Another example is developed by Ethicon, Surgifoam^®^ for medical applications of stopping the bleeding with the sponge-like highly absorbable properties [199].

Although there has been a great selection of sponge/foam-like hemostatic materials for bleeding control, some shortcomings associated with their *in vivo* applications need to be addressed in the fabrication of the next generation of hemostatic sponges. For instance, the degradation time of some of the commercially available absorbable sponges, such as those fibrin-based and gelatin-based hemostats, is very long-ranging from a few weeks to 12 months. Such a long biodegradation time may interfere with wound healing, resulting in scar formation or enhanced inflammatory responses [200]. In addition, some hemostatic sponges cannot withstand blood flow and may relocate, resulting in unwanted off-target adhesion to the surrounding tissues [201]. Furthermore, several pathological abnormalities, such as fibrotic and necrotic tissues associated with long-term implantation of hemostatic sponges, are reported in the literature [201, 202]. Another common observation regarding hemostatic sponges is infection after implantation [200]. Therefore, engineering of hemostatic sponges with antibacterial properties is preferred. According to the shortcomings mentioned above, their usage conditions should be well-understood to avoid unnecessary danger in clinical applications.

### Films and sheets

Increasing demands for innovative approaches are necessary to avoid excessive bleeding in different cases, including surgery, battlefield injuries and accident cases [1, 203–206]. Most successful hemostasis depends on patients' clotting capabilities. As a result, the medication efficacy may vary, or hemostatic medications sometimes fail [207]. While considering the different forms of hemostats or coagulant-loaded hemostats like hydrogel, foams, particles and sheets to stop the bleeding in the injury site [116, 150, 208, 209]. Hemostatic sheets or pads are a great candidate for ease of use and large-scale preparation without any laborious procedures [116]. In this section, we summarize the significance of hemostatic pads or sheets for hemorrhage control in different ways. Generally, hemostatic sheets are prepared with different types of natural or synthetic polymers [210]. They can be engineered in various forms, such as film sheets, nanofiber sheets and layer-by-layer approaches [211–213]. Topical hemostats like hemostatic sheets offer an advantage over other types of hemostats when controlling bleeding with vessel occlusion pressure or ligature methods is complicated, as is the case with wounds from which blood continuously seeps [211–213].

Due to their unique features, like absorption and adhesiveness, gelatin, chitosan and other naturally abundant biopolymers have been widely employed as hemostatic dressings [214]. Li *et al.* [211] prepared chitosan and gelatin films by solvent casting to create ibuprofen-loaded composite films to maintain sterility and control the bleeding in the surgical site. Tensile strength and elongation at break were influenced by the amount of chitosan in the composite films. The glutaraldehyde crosslinking presumably increased moisture vapor transfer and composite film swelling. Ibuprofen-loaded chitosan/gelatin composite films outperformed the control in antibacterial activity tests against *E. coli* and *Staphylococcus aureus*. Furthermore, according to the assessment of the hemostatic effects, they could reduce bleeding in a surgical operation with low pressure and had good absorption properties.

Alginate has gained popularity as a hemostatic material due to its exceptional properties, such as wound adherence and water absorption [215, 216]. *Yunnan Baiyao*, a well-known herbal prescription used in oriental countries for over a century, has been shown to be safe and effective as a surgical sealant and hemostat [217]. Lu *et al.* [218] recently reported *Yunnan Baiyao* incorporated chitosan/alginate composite films for rapid coagulation in a rat liver hemorrhage model. Their results suggested that compared to controls, *Yunnan Baiyao* functioned synergistically with chitosan and sodium alginate to induce stronger hemostasis.

Recently, bioactive materials have gained much attention in bone regeneration and wound healing [219–221]. Among them, mesoporous bioactive glass (MBG) is a new class of material that offers consistent nanoscale mesoporous structure with high-specific surface areas and good biological activity [221]. Recently, Jia *et al.* [222] prepared an MBG/chitosan hemostatic film via solvent casting, followed by freeze-drying. The MBG/chitosan films showed good water adsorption and were tuned by changing the MBG/chitosan composite ratio during preparation. Different MBG/chitosan ratio composite films demonstrated varying hemostatic effects in a rat hepatic hemorrhage model. The hemostatic time and volume of bleeding decreased with MBG. The composite films were easily degradable *in vitro*, were biocompatible and non-cytotoxic, indicating that MBG/chitosan composite porous films function as a novel hemostatic material compared to previous hemostatic materials.

Sometimes technically improved hemostats are necessary to enhance the efficacy and functions [116, 223]. For example, Takagi *et al.* [116] recently reported a gelatin-based two-layer sheet composed of gelatin sponge and gelatin sheet as a topical agent to stop bleeding. They compared their material with commercially available hemostats TachoSil^®^ made by collagen and loaded with clotting molecules like thrombin and fibrinogen. Their results showed that the two-layer gelatin sheet was a better topical hemostatic agent than TachoSil^®^ with a lesser danger of viral transmission and inflammation than topical treatments containing fibrin components and/or thrombin. Their two-layer gelatin sheets showed excellent hemostatic efficacy in bleeding dog spleens (an organ where bleeding is difficult to control) and other organs, such as the liver, kidney, lung and arteries. Lately, microneedle technology has shown great promise in various applications, including drug and cell delivery [224, 225]. Inspired by that, Yokoyama *et al.* [223] developed a hemostatic sheet based on their microneedle technology. In addition, their microneedle hemostatic sheet can be potentially used in intracorporeal topical hemostasis for parenchymatous organs, major arteries and the heart wall following trauma or surgery.

**Figure 7.**
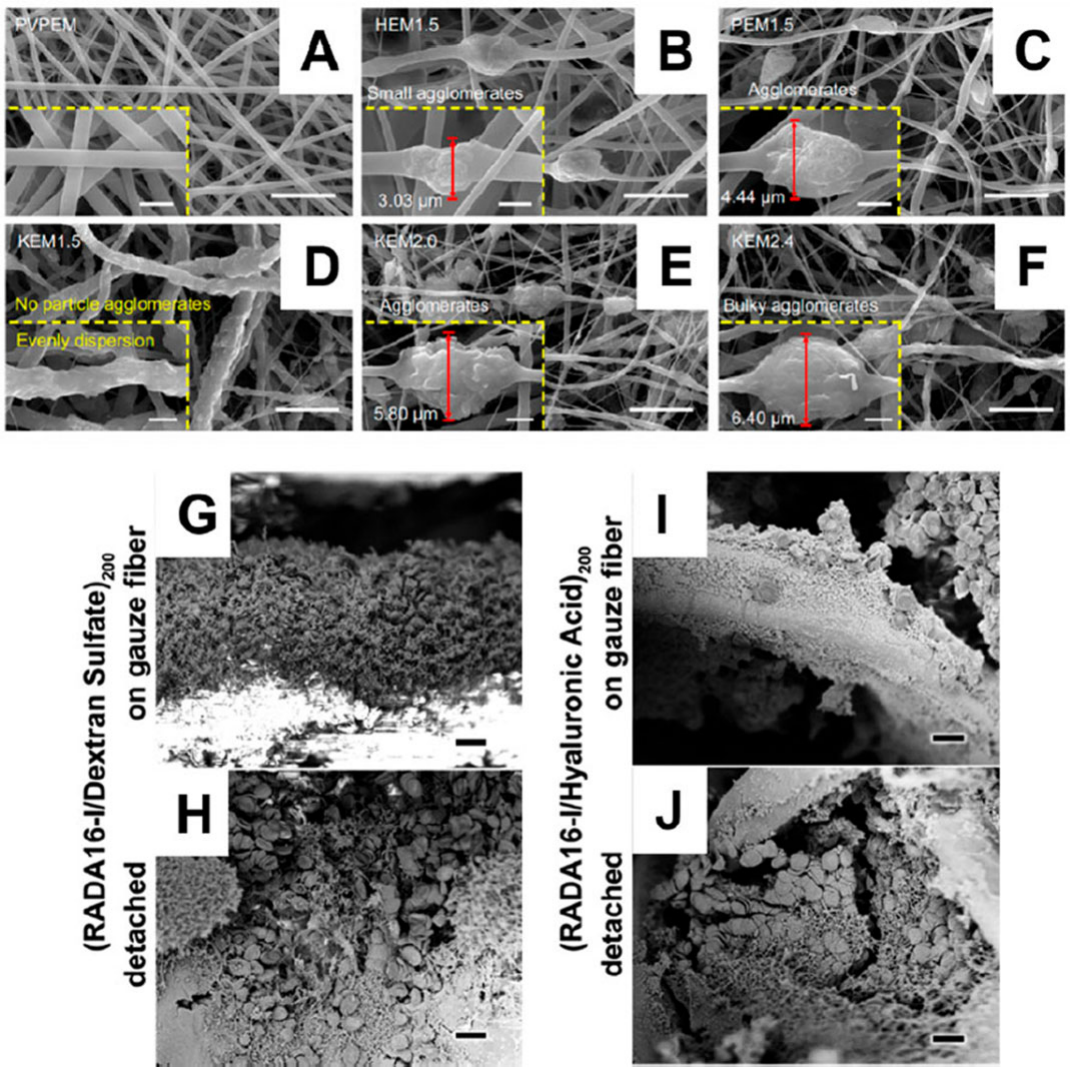
SEM images of fiber sheet and film hemostats. (**A**) PVP electrospun membranes, (**B**) halloysite electrospun membranes (1.5 g nanoclay), (**C**) palygorskite electrospun membranes (1.5 g nanoclay), (**D**) kaolinite electrospun membrane (1.5 g nanoclay), (**E**) kaolinite electrospun membrane (2.0 g nanoclay) and (**F**) kaolinite electrospun membrane (2.4 g nanoclay). The insets are the corresponding 5× magnification of the images (scale bar = 2 µm). Images adapted with permission from Cui *et al.* [228]. SEM images of anticoagulated whole blood in contact with (RADA16-I/dextran sulfate) 200 films (**G** and **H**) and (RADA16/HA) 200 films (**I** and **J**) show interaction of the films with the blood components (scale bar = 10 µm). Images adapted with permission from Hsu *et al*. [213].

Nanofibrous sheets exhibit similar morphology to the natural extracellular matrix, with varying pore size distribution and large surface areas [212, 62]. Therefore, nanofibrous sheet hemostats can be a great candidate compared to film-based hemostats. Generally, nanofibrous sheets are prepared using different techniques, like electrospinning, and self-assembly [226, 227]. Among them, electrospun nanofibrous sheets have gained much attention due to their simplicity and large-scale production [226]. For example, Cui *et al.* [228] recently reported a nanoclay-based polyvinyl pyrrolidine (PVP) hemostatic nanofiber membranes (Fig. 7A–F). The nanoclay electrospun membrane (NEM) with Kaolinite (KEM) showed 60% higher hemostatic performance than other NEMs in both *in vitro* and *in vivo* models. The influence of extracellular matrix microstructure and nanoclay (60%) played a pivotal role in reducing clotting time, suggesting that KEM bandages are a good candidate for compressible hemorrhage management. In another study, Gu *et al.* [62] reported electrospun-chitosan/gelatin nanofibrous sheets with improved pore size and surface area via ultrasonic treatment. Both hydrophilic gelatin and ultrasonication had synergetic effects on hemostatic activities, such as quick blood absorption and coagulation *in vitro*. Furthermore, it also encouraged active cell growth, migration and invasion. These results suggested that sonicated chitosan/gelatin nanofiber mats could be used as a hemostatic dressing and tissue engineering scaffold.

In severe cases, hemostatic substances in the form of fibers and non-woven sheets can be applied and withdrawn quickly from the bleeding site [229]. Fiber and non-woven dressings have excellent permeability and an extremely porous microstructure and have been widely used for wiping blood from surgical sites. Interestingly, a biodegradable and biocompatible polycaprolactone (PCL) with chitosan fibrous sheet was prepared using electrospinning and modified drop-casting [212]. The chitosan/PCL nanofibrous sheets were stronger than chitosan/PCL blended nanofibers and just the chitosan membrane alone. Moreover, they reported that adding tree tea oil to the chitosan overlayer of chitosan/PCL dressing increased platelet aggregation, antibacterial and anti-inflammatory characteristics.

ORC is an absorbable hemostatic material used in surgery to control low-pressure and diffuse bleeding [230]. He *et al.* [78] developed a water-soluble chitosan/ORC composite gauze produced using an *N, O*-carboxymethyl chitosan (*N, O*-chitosan)-modified version of ORC gauze. *N, O*-chitosan/ORC composite gauze had excellent hemostatic properties in a rabbit liver bleeding model. Furthermore, its antibacterial effectiveness increased as the *N, O*-chitosan percentage increased. These findings demonstrated that *N, O*-chitosan/ORC composite gauze could be an effective hemostatic device and adhesion prevention tool after surgery.

In another study, Jaiswal *et al.* [231] reported the hemostatic efficacy of PCL/gelatin nanofibrous matrix in rat livers. The time to stop bleeding, prothrombin time and fibrinogen concentration were all measured, and showed that the electrospun sheets caused fast hemostasis in all test animals. They also examined the livers of both treated and untreated mice and found that the test group's liver wounds healed faster than the control group. The nanofibrous matrix showed potential for liver regeneration and safety and efficacy as a hemostat.

Similarly, Li *et al.* [232] reported curcumin-loaded mesoporous silica incorporated antibacterial PVP nanofibers. The engineered mats were tested for structure, biocompatibility, antibacterial activity and hemostatic impact in an *in vivo* liver injury model. A loading ratio of <8% CCM-MSN in PVP electrospun nanofibers was found to be homogenous. The hybrid nanofiber mats were non-cytotoxic and more effective against methicillin-resistant *S. aureus* (MRSA) *in vitro*, which was validated via *in vivo* tests. When exposed to blood, the hybrid nanofiber mats quickly change into hydrogels, activating the clotting system to stop the wound from bleeding. With their excellent biocompatibility and antibacterial activity, CCM-MSN integrated PVP nanofiber mats are a suitable option as a hemostatic material.

The Hammond group has developed thin-film coatings on gauze and gelatin sponges using layer-by-layer technology to function as hemostatic devices [213, 233]. These nanofibers elute RADA-16 peptide upon hydration and form nanofiber-based clots when they come in contact with blood (Fig. 7G–J). Moreover, compared to ordinary gauze, these nanofiber-based films from gauze bandages accelerated hemostasis in porcine skin lesions. Due to RADA-16 peptide's potent hemostatic activity, biocompatibility, biodegradability and low production costs, their strategy is a promising cheap yet effective hemostatic bandage.

## Conclusions and future perspectives

In this review, we first describe the coagulation cascade, which involves numerous pathways to stop bleeding, followed by a discussion of the effects of clotting factors in hemostasis. Next, we summarize the biopolymer-based hemostats covering major polysaccharides- and polypeptides-based materials for hemostatic applications fabricated into various engineered forms, such as powder, sponges, sheets and hydrogels.

From a materials design point of view, chemically modified biopolymer-based hemostats with strong adhesion to tissue will need to be developed for passive hemostasis, without activating the coagulation process or causing systemic embolism and thrombosis. Compared to synthetic polymers, the mechanical properties of biopolymer-based hemostatic agents are weak. Therefore, developing advanced biofabrication techniques will be necessary to engineer biopolymers into clinically effective and mechanically robust hemostatic materials. For example, structurally reinforced foams or aerogels could be designed to help pressure dissipation at the wound site for non-compressible hemorrhages. Moreover, the addition of bioactive hemostatic additives, such as silica nanoparticles, into the biopolymer-based substrates has also been reported to trigger the coagulation cascade to improve the hemostatic ability of materials.

In terms of clinical translation, surgeons are demanding the development of high-quality hemostats with rapid blood clotting capacity with ease of use under any conditions. Therefore, researchers and developers in the field of hemostats and sealants, as well as adhesives, are continually motivated by the desires of surgeons for improved tools that will make it easier for them to perform technically challenging operations. In the case of topical injuries, several research efforts have been reported on various natural and synthetic biomaterials. Specifically, most hemostats exhibit different forms, including adhesive, band power, foams and gels. Table 3 shows the biopolymer-based hemostats on the market and are currently in National Institutes of Health (NIH) clinical trials.

**Table 3.** Biopolymer-based hemostats currently on the market and in National Institutes of Health (NIH) clinical trials

Product name	Main component(s)	Form of hemostat	Company/sponsor	Phase	References
Floseal^®^	Gelatin matrix with thrombin	Gel Sealant	Baxter	On-market	[234,235]
Surgicel^®^	ORC	Powder	Ethicon	On-market	[236]
Surgifoam^®^	Gelatin	Sponge/powder	Johnson and Johnson	On-market	[237, 238]
Celox Granules	Chitosan	Powder	Celox Medical	On-market	[239, 220]
Spongostan™	Gelatin	Sponge-like dressing	Ethicon	On-market	[240]
BaneCel^®^	Oxidized cellulose	Gel Sealant	Unicare Biomedical	On-market	[241]
HemCon^®^ Bandage PRO	Chitosan	Sponge-like dressing	Tricol Biomedical	On-market	[242]
Avitene™	Collagen	Sheet/sponge	BD	On-market	[243]
Gelfoam^®^	Gelatin	Sponge	Pfizer	On-market	[244]
Tachosil^®^	Fibrin	Patch	Baxter	On-market	[245, 246]
Surgiflo^®^	Gelatin matrix with thrombin	Gel Sealant	Ethicon	On-market	[234, 247]
PeproStat	Recombinant human albumin (rHA) conjugated with fibrinogen-binding peptides	Liquid	Haemostatix Ltd	Phase 2 (NCT03131336)	[248]
Evarrest^®^	Fibrin	Patch	Ethicon	Phase 3 (NCT03255174)	[249]
Thrombi-Gel^®^	Thrombin-JMI (bovine-derived), carboxylmethylcellulose	Sponge/pad	Vascular Solutions LLC	Phase 4 (NCT00652314)	[250, 251]
	Gelatin				
HEMOCOLLAGENE^®^	Collagen	Sponge	Septodont	N/A	[252]
				(NCT05171231)	

Overall, challenges remain in developing next-generation biopolymer-based hemostatic agents. Scientists with a background in materials sciences, engineering, biology, clinics and even electronics engineering, need to join forces in exerting the potential to advance naturally derived materials into desired hemostats. Furthermore, intelligent skin-interfaced electronics is another emerging topic that should be explored to achieve smart monitoring of the coagulation process and responsive therapeutics to the wound sites at the same time.
